# Deprescribing preventive medications in older adults with advanced frailty, dementia, or limited life expectancy: a systematic review and meta-analysis

**DOI:** 10.1186/s12877-026-07354-5

**Published:** 2026-03-17

**Authors:** Saibal Das, Vrushali Shendage, Tanay Maiti, Mihir Bhatta, Sarnendu Mondal, Rajesh Aadityan, Deepasree Sukumaran, Sanskriti Shukla, Ashish Pathak, Samiran Panda, Santanu Kumar Tripathi, Cecilia Stålsby Lundborg

**Affiliations:** 1 Department of Global Public Health, Karolinska Institutet, Stockholm, Sweden; 2 Indian Council of Medical Research - National Institute for Research in Bacterial Infections, Kolkata, India; 3 Academy of Scientific and Innovative Research (An Institution of National Importance Established by an Act of Parliament), Ghaziabad, India; 4 Department of Epidemiology and Global Health, Umeå University, Umeå, Sweden; 5 South West Yorkshire NHS Foundation Trust, Wakefield, UK; 6 All India Institute of Medical Sciences, Raebareli, India; 7 Department of Pharmacology, All India Institute of Medical Sciences, Kalyani, India; 8 Translational Health Science and Technology Institute, Faridabad, India; 9 Department of Pediatrics, RD Gardi Medical College, Ujjain, India; 10 Indian Council of Medical Research, New Delhi, India; 11 Jagannath Gupta Institute of Medical Sciences and Hospital, Kolkata, India

**Keywords:** Deprescribing, Frailty, Older adults, Preventive medications, Polypharmacy

## Abstract

**Objective:**

Polypharmacy and long-term preventive medication use are common in frail older adults with limited life expectancy, despite uncertain benefits and potential risks. This systematic review and meta-analysis synthesized evidence on the effect of deprescribing preventive medications (antihypertensives, statins, anticoagulants, and antidiabetic agents) compared to continuation on clinical, physiological, safety, and patient-centered outcomes among older adults with advanced frailty, dementia, or limited life expectancy.

**Methods:**

PubMed, Embase, Cochrane Library, Web of Science, CINAHL, and ProQuest Dissertations & Theses Global were searched for eligible randomized controlled trials and observational studies. The primary outcome was all-cause mortality. Secondary outcomes were hospitalization, major adverse cardiovascular events (MACE), changes in blood pressure, risks of fractures and falls, and quality of life. Data were pooled (relative risk [RR] or mean difference or standardized mean difference) using random-effects models (RevMan version 5.4). The evidence certainty was evaluated by the GRADE framework (PROSPERO ID: CRD420251147086).

**Results:**

From 10,397 records, 15 studies (> 33,000 participants) were included. Overall, deprescribing was not associated with increased risk of all-cause mortality (RR: 1.15, 95% CI: 0.98–1.35, I^2^: 93%), hospitalization (RR: 0.93, 95% CI: 0.82–1.07, I^2^: 68%), or MACE (RR: 1.37, 95% CI: 0.70–2.70, I^2^: 95%) (certainty: very low GRADE). Deprescribing was also not associated with increased risks of fracture, fall, or deterioration of quality of life, but with slightly increased systolic blood pressure (deprescribing antihypertensives).

**Conclusion:**

Deprescribing preventive medications in frail or palliative older adults was not associated with worse outcomes; however, evidence certainty was very low, and further studies are needed.

**Supplementary Information:**

The online version contains supplementary material available at 10.1186/s12877-026-07354-5.

## Introduction

Polypharmacy has become an increasingly prevalent challenge in aging populations, particularly among older adults living with multimorbidity, frailty, and limited life expectancy [1]. As the average lifespan extends, a growing proportion of individuals spend their final years with multiple chronic conditions managed by complex drug regimens [2]. Preventive medications, such as antihypertensives, statins, anticoagulants, and antidiabetic agents, are routinely prescribed to reduce the long-term risk of cardiovascular and metabolic complications [3]. While these agents offer clear benefits in younger, healthier populations, their advantages diminish in advanced age and illness, when the time to benefit exceeds remaining life expectancy. At the same time, the potential for harm, including falls, bleeding, hypotension, hypoglycemia, drug-drug interactions, and treatment burden and cost, rises substantially [4].

In such contexts, deprescribing, i.e., the planned, supervised process of dose reduction or discontinuation of medications that are no longer beneficial or may be causing harm, has emerged as an essential strategy to optimize pharmacotherapy [5]. It aligns treatment with the changing goals of care at the end of life, emphasizing comfort, function, and quality rather than long-term disease prevention [6] Deprescribing is now recognized as a component of good clinical practice in geriatrics and palliative medicine [7]. However, despite increasing advocacy, clinicians often hesitate to withdraw preventive drugs due to uncertainty regarding clinical outcomes, patient and family expectations, and a lack of high-quality evidence to support safety [8].

The clinical and ethical importance of deprescribing in this vulnerable population warrants a comprehensive evaluation of clinical, physiological, safety, and patient-centered outcomes across diverse drug categories and care settings. Accordingly, this systematic review and meta-analysis aimed to synthesize and appraise evidence from the literature investigating deprescribing of preventive medications among frail older adults, including those with advanced dementia, severe comorbidity, or a life expectancy of less than one year. By integrating data across multiple drug classes and clinical settings, this study provides an updated, evidence-based foundation for informed deprescribing decisions in late-life care.

## Methods

### Protocol and registration

The review followed the PRISMA 2020 guidelines. The protocol was registered in PROSPERO (CRD420251147086).

### Study selection criteria

Randomized controlled trials (RCTs) and observational studies that included adults aged ≥ 60 years with advanced frailty, dementia, or limited life expectancy in hospital, hospice, nursing-home, or community palliative settings and studied deprescribing, tapering, or de-intensification of preventive medications (antihypertensives, statins, anticoagulants, and antidiabetics) were included. Frailty definitions varied across studies, and clinically vulnerable older populations with multimorbidity, cognitive impairment, or functional limitations were considered consistent with frailty-related vulnerability in geriatric populations. These medication classes were selected a priori as long-term cardiometabolic therapies prescribed primarily for risk reduction rather than symptom control. They were chosen because they are among the most frequently prescribed chronic medications in older adults, are commonly continued in the context of frailty and limited life expectancy, and have well-described time-to-benefit profiles that may exceed remaining life expectancy, making them particularly relevant to deprescribing decisions. The comparators were continuation or usual care. Studies including older adults without frailty or limited life expectancy, observational studies without any comparator group, cross-sectional prevalence/predictor of deprescribing studies, and feasibility studies were not included. Case reports, case series, editorials, qualitative studies, and preprints were also not included.

### Search strategy

A literature search was performed in the following databases: PubMed, Embase, Cochrane Library, Web of Science, CINAHL, and ProQuest Dissertations & Theses Global from inception till 3 Oct 2025 without any language restriction. The search strategy was developed in collaboration with librarians at the Karolinska Institutet University Library. For each search concept, Medical Subject Headings (MeSH-terms) and free text terms were identified. The search was then translated, in part using Polyglot Search Translator [9], into the other databases. The strategies were peer reviewed by another librarian prior to execution. The full search strategies for all databases are available in Table s1.

### Study screening

De-duplication of the studies was done using Covidence. Two groups of independent authors (SD, TM, MB, and SM) reviewed the titles and abstracts of the identified studies. Subsequently, the authors retrieved the abstracts and, if necessary, the full texts of the articles to assess their suitability for inclusion. The web-based Rayyan software was used for this purpose. To obtain any missing information, the corresponding authors of the relevant articles were contacted via email. Only articles with full-text access were considered for inclusion in the study. Conference proceedings, review articles, commentaries, and similar publications were not included in the analysis. A senior author (DS) was consulted for disagreement resolution.

### Risk of bias assessment

The included studies were assessed for risk of bias using the revised Cochrane risk-of-bias 2 tool [10] for RCTs and the ROBINS-I tool for observational studies [11].

### Data synthesis

During the abstract reviewing and data extraction process, three authors (SD, VS, and RA) independently conducted the tasks using a pre-formatted data extraction spreadsheet. No assumptions or simplifications were made during the data extraction process to ensure accuracy and reliability. Summary estimates were utilized for the analysis, and meta-analysis was conducted whenever there was sufficient data available for the pre-defined primary and secondary outcomes. The statistical analysis was performed using RevMan version 5.4 software. A random-effects model (DerSimonian-Laird) was employed to ensure the robustness of the model across different populations and to account for potential outliers. The relative risk (RR) with a corresponding 95% confidence interval (CI) was used to estimate the pooled treatment effects for the dichotomous outcomes, and the mean difference or standardized mean difference with a corresponding 95% CI was used to estimate the pooled treatment effects for the continuous outcomes. In cases where data were insufficient, descriptive statistics were utilized. Attrition rates, including dropouts, loss to follow-up, and withdrawals, were also investigated during the analysis. Issues of missing data and imputation methods were critically appraised, and the intention-to-treat data were used for the analysis of RCTs [12]. Heterogeneity was analyzed using the *χ*^*2*^ test on n-1 degrees of freedom, with an *α* error of 5% used for statistical significance, and with an I^2^ test [13]. The I^2^ values of 25%, 50%, and 75% corresponded to low, medium, and high levels of heterogeneity, respectively. Publication bias was assessed visually via funnel plots.

### Outcomes

The primary outcome was all-cause mortality. Secondary outcomes were hospitalization, major adverse cardiovascular events (MACE), including thromboembolic events, changes in blood pressure, risks of fractures and falls, and quality of life.

### Certainty of evidence

The GRADE approach (Grading of Recommendations Assessment, Development and Evaluation) [14, 15] was used to assess the certainty of evidence.

## Results

**Fig. 1:**
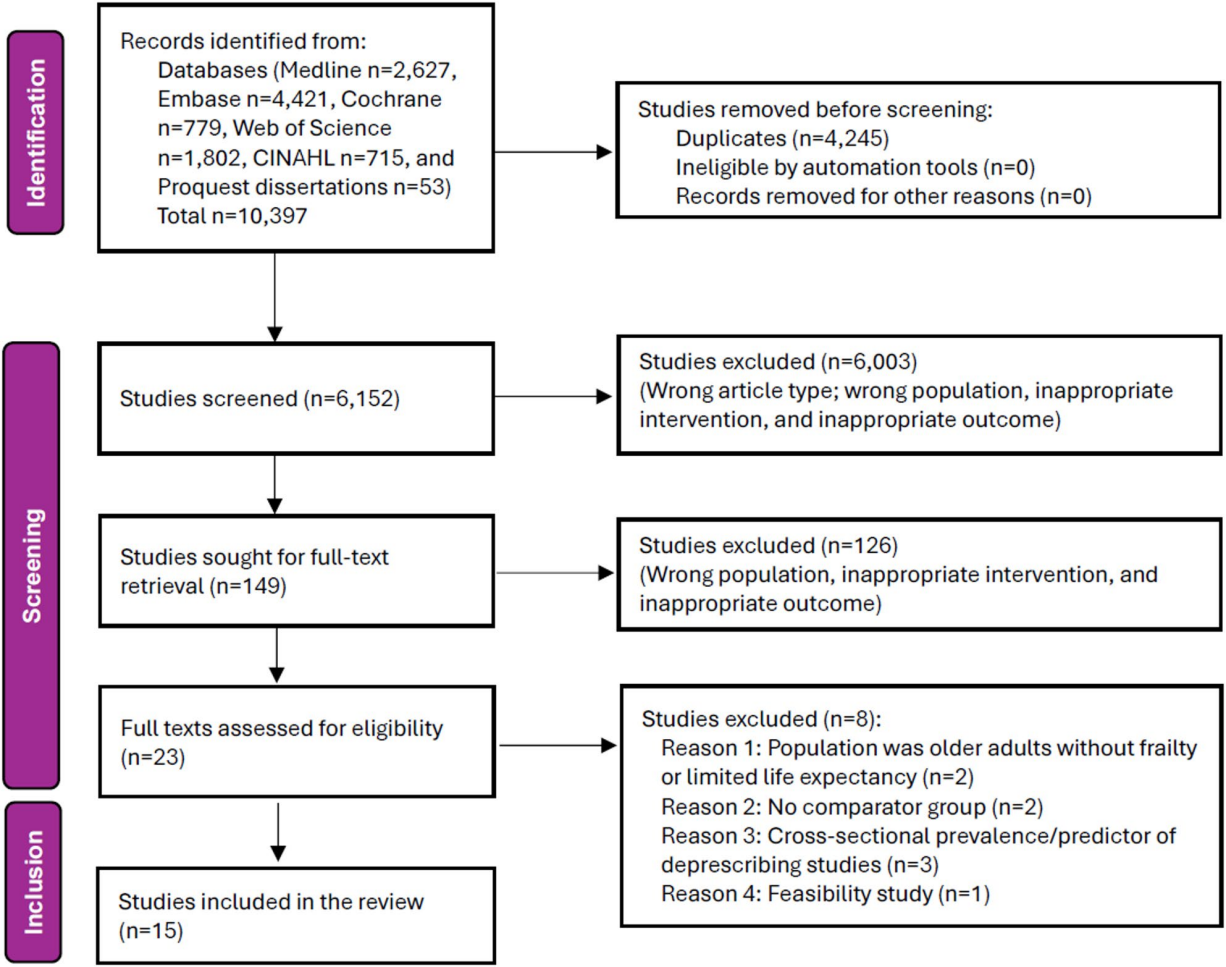
PRISMA flowchart

The initial database search yielded a total of 10,397 records. After removing duplicates, 6,152 titles and abstracts were screened, of which 23 full-text articles were assessed for eligibility. Ultimately, 15 studies [16–30] fulfilled all inclusion criteria and were included in the quantitative and qualitative synthesis. The PRISMA flowchart describing the selection process is presented in Fig. 1. The included studies comprised ten RCTs [16], [19–21], [24–26], [28–30] and five observational studies [17, 18, 22, 23, 27] published between 2015 and 2025, representing a diverse range of settings, including hospital, hospice, nursing-home, or community palliative care facility. Among the ten included RCTs, five were judged to have a low overall risk of bias, and five had a moderate risk of bias (Table s2). Of the observational studies, three were assessed as having a moderate risk of bias, one as low risk, and one as serious risk of bias (Table s3). Together, these 15 studies involved > 33,000 participants with mean ages ranging from 65 to ~ 95 years. Ten studies had a higher female population than male. The majority of the participants were frail older adults with advanced multimorbidity, dementia, or terminal illness. Six [16, 20, 21, 25, 29, 30] of the studies evaluated the deprescribing of antihypertensive agents, two [22, 24] investigated statin discontinuation, three [17, 18, 23] focused on anticoagulant therapy, one [27] examined antidiabetic medication withdrawal, and three [19, 26, 28] assessed multiple medication deprescribing interventions. Follow-up periods varied between 16 weeks and six years, allowing both short-term and medium-term outcomes to be captured. Sex-wise outcome data were not reported in the individual studies. The characteristics of the included studies are enumerated in Table 1.

**Table 1:** Characteristics of the included studies

Author, Year	Country	Study design	Population studied	Age (years) (mean±standard deviation)	Medication class studied	Sample size used for analysis (intervention arm, comparator arm)	Type of deprescribing intervention	Comparator	Duration of follow-up
Benetos, 2025 [16]	France	RCT	Nursing-home residents of age ≥ 80 years with frailty on ≥ 2 antihypertensives and SBP of < 130 mm Hg	Intervention: 90.0 ± 4.8 Comparator: 90.1 ± 5.3	Antihypertensives	528, 520	Stepwise deprescribing of antihypertensives guided by protocol	Usual care	Median: 38.4 months (IQR: 30.0–48.0)
Bertozzo, 2016 [17]	Italy	Prospective cohort	Patients aged ≥ 80 years on warfarin having non-valvular atrial fibrillation and maintaining an INR of 2–3	Intervention: 84.7 ± 3.4 Comparator: 84.3 ± 3.2	Anticoagulants (warfarin)	148, 650	Deprescribing of warfarin	Continuation of warfarin	Median: 26 months (IQR: 15–42)
Chin-Yee, 2028 [18]	Canada	Retrospective cohort	Older adults aged ≥ 66 years in home palliative care on anticoagulants	Intervention: 81.2 ± 8.2 Comparator: 81.2 ± 8.2	Anticoagulants (VKA, DOACs)	1990, 6166	Deprescribing of anticoagulants after palliative care initiation	Continuation of anticoagulants	Median: 111 days (IQR: 32–400)
Curtin, 2020 [19]	Ireland	RCT	Older adults aged ≥ 75 with advanced frailty and polypharmacy and transferring to long-term nursing home care	Intervention: 84.49 ± 5.60 Comparator: 85.68 ± 5.87	Multiple preventive medications	51, 47	STOPPFrail deprescribing intervention	Usual care	3 months
Gulla, 2018 [20]	Norway	Cluster RCT (COSMOS sub-study)	Patients ≥ 65 years old with frailty	Intervention: 86.9 ± 7.6 Comparator: 87.5 ± 7.2	Antihypertensives	164 (35 units), 131 (31 units)	Deprescribing of antihypertensives by systematic medication review (collegial mentoring)	Usual care	9 months
Husebø, 2019 [21]	Norway	Cluster RCT (COSMOS study)	Patients ≥ 65 years old with frailty	Intervention: 86.5 ± 7.7 Comparator: 87.0 ± 7.2	Antihypertensives	297 (36 units), 248 (31 units)	Deprescribing of antihypertensives by multicomponent intervention (standardized education and training)	Usual care	9 months
Ioffe, 2021 [22]	Israel	Retrospective cohort	Patients aged ≥ 80 years and hospitalized	Intervention: 87.1 ± 4.5 Comparator: 83.6 ± 3.0	Statins	140, 229	Deprescribing of statins at admission	No statin use	12 months
Kempers, 2025 [23]	The Netherlands	Retrospective cohort	VKA users with life-limiting disease or cancer	Overall: 80.3 ± 8.9	Anticoagulants (VKA)	2937, 14,208	Deprescribing of anticoagulants within 1 year of diagnosis	Continuation of anticoagulants	Median: 3.6 years
Kutner, 2015 [24]	USA	RCT	Older adults with advanced, life-limiting illness with an estimated life expectancy of 1 month to 1 year	Intervention: 74.8 ± 11.7 Comparator: 73.5 ± 11.5	Statins	182, 189	Deprescribing of statins	Continuation of statins	1 year
Moonen, 2016 [25]	The Netherlands	RCT (DANTE Study)	Community-dwelling older adults of age ≥ 75 years with mild cognitive deficits and on antihypertensives	Intervention: 81.1 ± 4.3 Comparator: 81.5 ± 4.6	Antihypertensives	180, 176	Deprescribing of antihypertensives	Continuation of antihypertensives	16 weeks
Mortsiefer, 2023 [26]	Germany	Cluster RCT (COFRAIL study)	Community-dwelling older adults of age ≥ 70 years with frailty, on polypharmacy, having life expectancy of ≥ 6 months, and without moderate or severe dementia	Intervention: 83.69 ± 6.08) Comparator: 83.29 ± 6.29	Multiple preventive medications	262, 248	GP-based deprescribing aided by training, guideline, and tool-kit	Usual care	12 months
Niznik, 2022 [27]	USA	Retrospective cohort	Newly admitted older nursing-home residents with advanced dementia or limited life expectancy and potentially overtreated for diabetes	Intervention: 65–74: 38.3% 75–84: 42.4% ≥ 85: 19.3% Comparator: 65–74: 38.3% 75–84: 40.6% ≥ 85: 21.1%	Antidiabetics	554, 1528	Deintensification of antidiabetics	Continuation of antidiabetics	6 years
Potter, 2016 [28]	Australia	RCT	Older adults living in residential aged care facilities with frailty	Intervention: 84 ± 6 Comparator: 84 ± 6	Multiple preventive medications	35, 32	Deprescribing intervention by medication review	Usual care	12 months
Sheppard, 2020 [29]	UK	RCT (OPTiMISE study)	Older adults of age ≥ 80 years, SBP of < 150, and on ≥ 2 antihypertensives	Intervention: 84.6 ± 3.3 Comparator: 85.0 ± 3.5	Antihypertensives	185, 269	Deprescribing of antihypertensives	Usual care	12 weeks
Sheppard, 2024 [30]	UK	RCT (follow up of OPTiMISE study)	Adults of age ≥ 80 years, SBP of < 150, and on ≥ 2 antihypertensives	Intervention: 84.7 ± 3.3 Comparator: 85.0 ± 3.6	Antihypertensives	280, 284	Deprescribing of antihypertensives	Usual care	Median: 4.0 years (IQR: 3.7–4.3).

*DOACs* direct oral anticoagulants, *INR* international normalized ratio, *IQR* interquartile range, *RCT* randomized controlled trial, *SBP* systolic blood pressure, *STOPPFrail* screening tool of older persons prescriptions in frail adults with limited life expectancy, *UK* United Kingdom; *USA* United States of America, *VKA* vitamin K antagonist

**Fig. 2:**
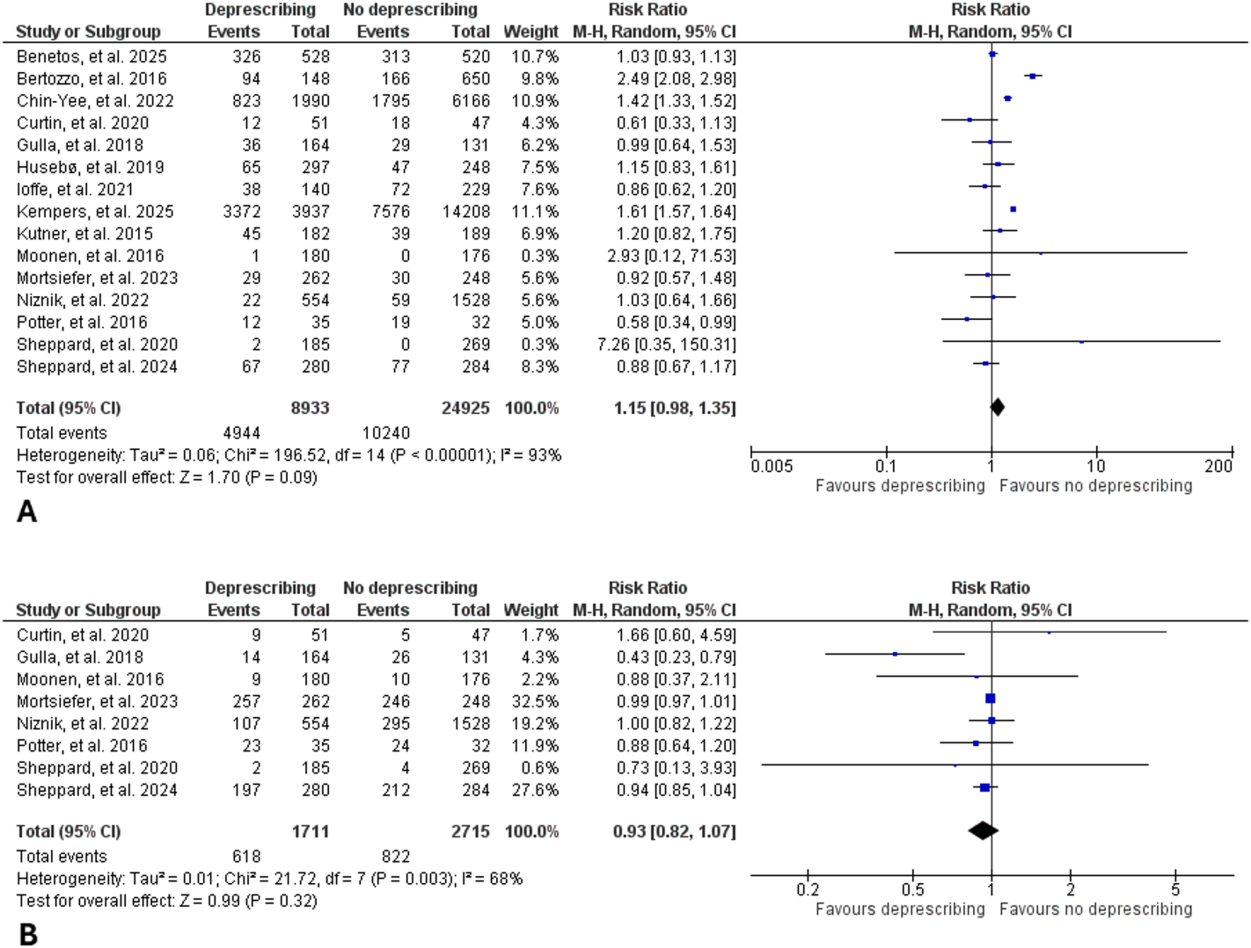
Forest plot showing the effect of deprescribing vs. medication continuation groups on the risks of mortality (**A**) and hospital admission (**B**)

**Fig. 3:**
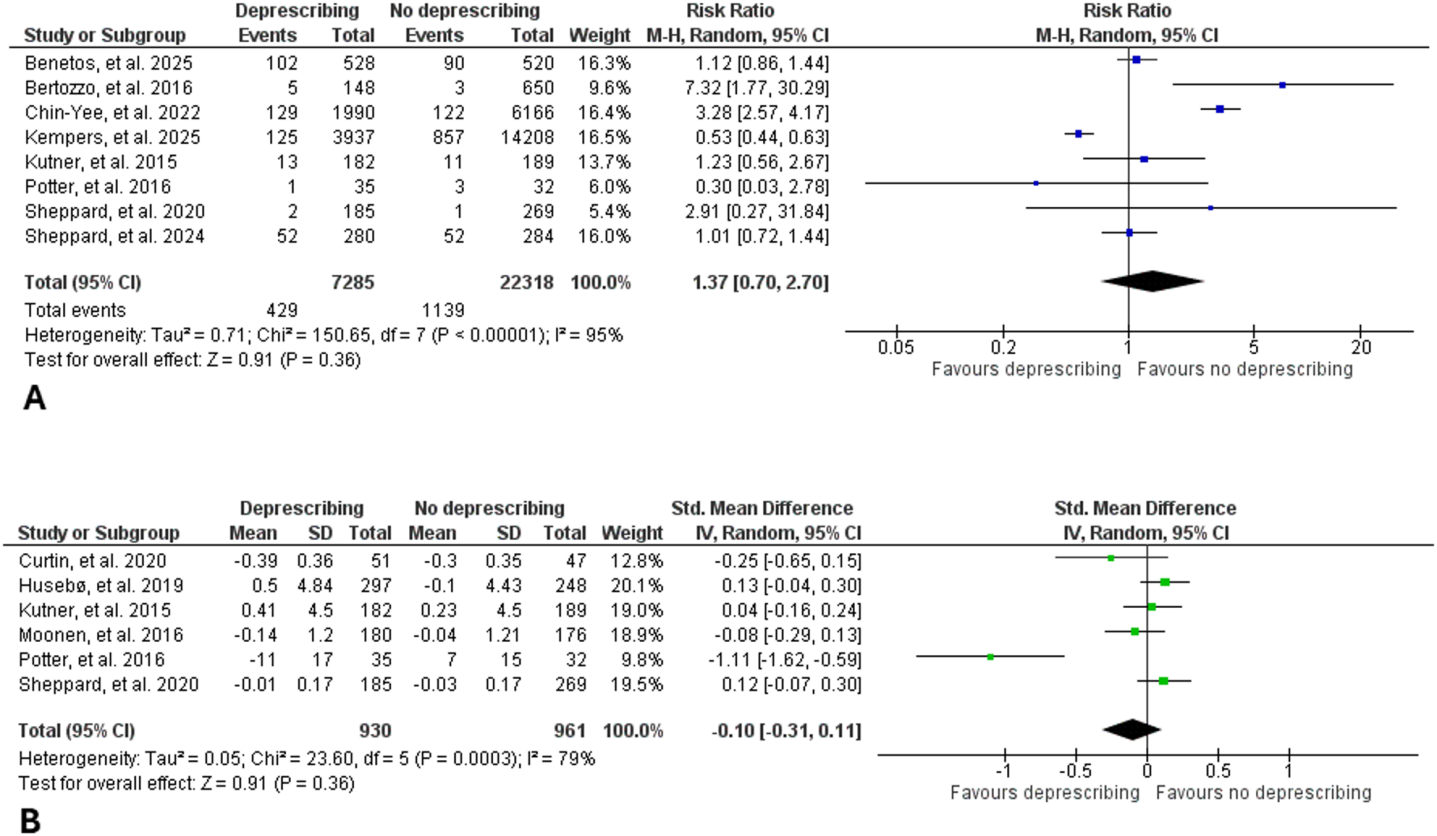
Forest plot showing the effect of deprescribing vs. medication continuation groups on the risk of major adverse cardiovascular events (**A**) and quality of life (**B**)

Regarding the primary outcome of all-cause mortality, pooled analysis across all included studies showed no significant difference between deprescribing and medication continuation groups (RR: 1.15, 95% CI: 0.98–1.35, I^2^: 93%), indicating that deprescribing did not increase mortality risk among frail or end-of-life populations (Fig. 2A). The results were consistent when only RCTs were included (RR: 0.98, 95% CI: 0.81–1.19, I^2^: 21%). Regarding the secondary outcomes, meta-analysis showed no statistically significant difference in the risk of hospital admission (RR: 0.93, 95% CI: 0.82–1.07, I^2^: 68%) (Fig. 2B), risk of MACE (RR: 1.37, 95% CI: 0.70–2.70, I^2^: 95%) (Fig. 3A), or quality of life (standardized mean difference: -0.10, 95% CI: -0.31-0.11, I^2^: 79%) (Fig. 3B) between deprescribing and continuation groups. Overall, deprescribing was not associated with increased risks of fracture (RR: 0.86, 95% CI: 0.59–1.25, I^2^: 5%) (Figure s1) or fall (RR: 0.97, 95% CI: 0.87–1.09, I^2^: 59%) (Figure s2). Deprescribing antihypertensives was associated with a slight increase in systolic blood pressure (mean difference: 4.90 mm Hg, 95% CI: 0.33–9.46, I^2^: 95%) (Figure s3) but no increase in diastolic blood pressure (mean difference: 1.48 mm Hg, 95% CI: -1.87–4.84, I^2^: 96%) (Figure s4). The certainty of evidence (GRADE) was very low for all-cause mortality, hospitalization, MACE, and quality of life outcomes (Table s4). Funnel plots showed no major evidence of publication bias (Figures s5-s7).

## Discussion

This systematic review and meta-analysis comprehensively evaluated the clinical effects of deprescribing preventive medications among frail and end-of-life older adults. Across fifteen studies, including ten randomized controlled trials and five observational studies, no significant difference was found in all-cause mortality, hospitalization, or MACE between deprescribing and continuation of preventive medications. Although some individual studies reported improvements in quality of life following structured deprescribing interventions, the pooled analysis did not demonstrate a statistically significant overall effect. Taken together, these findings support consideration of deprescribing long-term preventive medications in patients with limited life expectancy, emphasizing careful, individualized clinical judgment.

### Principal findings and interpretation

The principal outcome of this review, the absence of an increased mortality risk, has important clinical implications. Preventive medications, such as antihypertensives, statins, anticoagulants, and antidiabetics, are traditionally prescribed as they are proven to reduce long-term cardiovascular or metabolic risks [31]. However, their benefit-to-risk ratio changes markedly in frail populations with a shortened lifespan. Continuing these drugs may expose patients to adverse effects, complex medication regimens, and diminished quality of life, which can often be poor [32], without a realistic prospect of prevention of future disease events [33]. In our pooled analysis, mortality outcomes were consistent across studies with variable follow-up durations, suggesting that medication withdrawal in this population is unlikely to compromise survival.

The magnitude of effect across drug classes was remarkably consistent. Deprescribing antihypertensives produced a modest, clinically negligible rise in systolic blood pressure. These studies found that most participants maintained adequate blood pressure control after deprescribing, with no increase in cardiovascular events. Similarly, statin discontinuation yielded no survival disadvantage. For anticoagulants, our synthesis indicates that MACE events did not increase. This suggests that the overall risk-benefit profile remains acceptable when deprescribing is applied selectively to patients with low thrombotic risk. Likewise, for antidiabetic medications, deprescribing or dose reduction did not lead to clinically meaningful deterioration.

### Comparison with prior reviews in the literature

Earlier reviews on deprescribing among the elderly have consistently demonstrated its potential to reduce polypharmacy and potentially inappropriate medications. Reviews preceding 2024 largely emphasized pharmacist-led and clinician-supported interventions using Beers or STOPP/START criteria to improve prescribing appropriateness and safety [5]. However, heterogeneity in intervention design and settings limited their generalizability. The interventions most likely to be effective in optimizing medication use among elderly or end-of-life adults are those that are multifaceted, individualized, and multidisciplinary. The review demonstrated that structured medication reviews integrated with clinical judgment, when performed by interdisciplinary teams typically involving physicians, pharmacists, and nurses, produced the most consistent benefits across mortality, hospitalization, and quality-of-life outcomes [5] A recent meta-analysis consolidated evidence from RCTs in community-dwelling older adults, finding a modest but statistically significant reduction in total medications and moderate-certainty evidence of benefit, suggesting small individual yet meaningful population-level effects [7]. Another systematic review and meta-analysis demonstrated moderate-certainty evidence that deprescribing interventions decreased the use of potentially inappropriate medications and overall medication burden among community-dwelling older adults. Although the magnitude of effect at the individual level was small, the cumulative impact at the population level could be substantial, considering the widespread prevalence of polypharmacy use in this population [34]. Another meta-analysis including 32 RCTs demonstrated that compared to routine care, deprescribing interventions significantly improve clinical outcome indicators for older adults [35]. It was demonstrated in another systematic review that the current evidence indicates that hospital-initiated deprescribing strategies in older adults with advanced or life-limiting illness may reduce prescribing inappropriateness in the short term. However, well-designed trials with larger sample sizes and extended follow-up are required to establish the effectiveness and durability of deprescribing interventions in late-life populations [36]. Another systematic review indicated that deprescribing may confer measurable benefits in individuals with advanced or life-limiting conditions, particularly through reductions in medication burden and associated costs. Although no consistent evidence of significant harm was observed, a minority of patients experienced potential adverse effects, underscoring the importance of structured monitoring and clinical oversight [37]. It was indicated in another systematic review that deprescribing in older adults with life-limiting illness and limited life expectancy may enhance medication appropriateness and holds promise for improving selected clinical outcomes while reducing health care costs; however, the current evidence requires confirmation through more robust and adequately powered studies [38].

### Biological and clinical rationale

The observed safety of deprescribing can be explained by the pathophysiological context of frailty and advanced illness. Frail older adults frequently demonstrate age-related alterations in pharmacokinetics and pharmacodynamics, including reduced renal and hepatic clearance, decreased albumin binding, altered body composition, and impaired homeostatic regulation. These changes increase drug exposure variability and susceptibility to adverse drug reactions [39]. In parallel, frailty is characterized by diminished physiological reserve and increased vulnerability to stressors, limiting the capacity to tolerate medication-related harms. As competing mortality risks rise and life expectancy shortens, the probability of realizing long-term preventive benefits decreases, while the immediate risk of adverse effects becomes proportionally greater [40]. For instance, tight blood pressure or glucose targets may precipitate falls, syncope, or hypoglycemia, causing functional decline.^34^ Therefore, the modest elevations in systolic blood pressure or HbA1c observed after deprescribing are not harmful but may, in fact, represent a safer equilibrium for frail physiology [41]. Similarly, discontinuing statins may improve energy levels and gastrointestinal tolerance, thereby enhancing daily comfort [42]. Psychological relief from reducing pill burden and medical surveillance further contributes to perceived well-being [43]. These mechanisms collectively support deprescribing as a means to rebalance treatment goals toward symptomatic relief and dignity rather than preventive abstraction.

### Ethical and public health perspectives

Deprescribing also carries profound ethical implications. The act of withdrawing medication challenges both clinicians’ ingrained “prescribe to protect” mindset and patients’ expectations about medical care [44]. Yet the ethical principle of non-maleficence obliges clinicians to avoid harm, including harm from overtreatment. Rather than representing therapeutic abandonment, deprescribing constitutes an evidence-based shift from disease-centric to goal-oriented care. It aligns pharmacological treatment with patients’ current health priorities, typically comfort, functional independence, and dignity rather than long-term prevention of asymptomatic disease [45]. In this sense, deprescribing reflects the principle of “proportionate care,” ensuring that medical interventions are justified by their expected near-term benefits. From a public-health perspective, the growing prevalence of multimorbidity and polypharmacy among aging populations amplifies the relevance of deprescribing [46]. In this context, systematic deprescribing not only benefits individual patients but may also reduce healthcare costs, decrease medication errors, and improve system sustainability [47].

### Environmental perspectives

Apart from this, another aspect to be considered is the environmental effects of medication use. The healthcare industry is one of the main contributors to greenhouse gas emissions, and a considerable portion of this is attributed to pharmaceuticals, considering the manufacturing, packaging, distribution, and disposal of drugs, which are all processes that contribute to the carbon footprint [48]. The polypharmacy effect also adds to this, considering that more drugs are being manufactured, more waste is being generated, and more contamination is being caused by residues of drugs found in water systems [49]. Rational deprescribing, therefore, may also have a small effect on mitigating this, considering its potential to decrease the quantity of drugs being used, the waste being generated, and the greenhouse gases being emitted, all of which contribute to climate change, a threat to human health on a planetary scale [50].

### Strengths and reliability of the evidence

The main strength of this review lies in its methodological comprehensiveness. We conducted an exhaustive search across seven major databases without language or regional restrictions, ensuring a global evidence base. Dual independent screening and data extraction minimized selection bias, and inclusion of both randomized and observational studies captured the full continuum of evidence from tightly controlled to real-world contexts. The pooled sample of > 33,000 participants provides robust statistical power. Moreover, consistent effect directions across multiple outcomes and drug classes strengthen causal inference. The findings are therefore reliable and broadly generalizable to clinical populations of frail older adults in hospitals, primary care, and long-term care facilities.

### Limitations

Despite these strengths, several limitations should be acknowledged. First, the operational definition of frailty or limited life expectancy varied widely across studies. This heterogeneity may have introduced population variability, although it also enhances generalizability by reflecting real-world diversity. Second, follow-up durations varied considerably across the studies. While appropriate for assessing near-term safety, these periods may not capture delayed outcomes such as vascular events after discontinuing statins. These could lead to the high heterogeneity observed in most of the outcomes. Nevertheless, in end-of-life populations, longer follow-up may not be clinically relevant given the limited expected survival. Third, the deprescribing protocols varied across studies. Fourth, several studies exhibited a moderate risk of bias. Fourth, one included trial [25] enrolled older adults with mild cognitive impairment rather than overt dementia or explicitly defined limited life expectancy. However, this population represents a clinically vulnerable late-life group frequently overlapping with frailty phenotypes, and inclusion was considered consistent with the predefined eligibility framework [51–53]. Finally, the outcomes assessed were predominantly biomedical. Only a few trials measured patient-reported outcomes such as quality of life or treatment satisfaction, despite these being the most meaningful endpoints in palliative settings.

### Implications for clinical practice

The findings of this review offer actionable insights for clinicians, health systems, and policymakers. First, deprescribing should be recognized as an integral component of high-quality geriatric and palliative care rather than an optional or last-resort intervention. Systematic medication review, guided by tools such as STOPPFrail or the Medication Appropriateness Index, can help identify candidates for safe withdrawal. Interdisciplinary teamwork is crucial: collaboration among physicians, pharmacists, nurses, and family caregivers ensures comprehensive evaluation of each patient’s pharmacologic profile and care goals [5, 54]. Second, clinical guidelines should evolve to incorporate deprescribing recommendations alongside initiation criteria. For instance, antihypertensive management guidelines could specify thresholds for de-intensification in frail elders, acknowledging that tight control may be harmful. Similarly, cardiometabolic guidelines could endorse deprescribing statins in patients with a life expectancy of under one year or severe functional dependency. By embedding deprescribing triggers into disease-specific protocols, professional societies can normalize this practice and reduce clinician hesitation. Third, education and communication strategies must be enhanced. Clinicians often cite fear of adverse outcomes, medico-legal liability, or perceived patient resistance as barriers to deprescribing. Training programs in communication and shared decision-making can empower practitioners to discuss deprescribing confidently and empathetically. Patient information leaflets and decision aids explaining the potential benefits of deprescribing [5, 55] may further facilitate acceptance.

### Implications for research

Future research should build upon the foundations established here by addressing remaining evidence gaps. Pragmatic cluster-randomized trials embedded within healthcare systems could evaluate multi-drug deprescribing programs rather than single-class interventions, reflecting the complexity of real-world polypharmacy. Studies should incorporate mixed-methods designs that capture qualitative data on patient and caregiver experiences, including emotional responses to deprescribing. In none of the included studies was a sex disaggregated analysis performed. In future studies, it would be important to add such an analysis. Additionally, health-economic analyses are warranted to quantify cost savings from reduced medication use, hospitalizations, and monitoring requirements. Another priority is the development of validated outcome measures specific to deprescribing in end-of-life care. Traditional endpoints, such as mortality or hospitalization, may not fully capture patient-centred benefits. Composite measures incorporating symptom control, functional stability, satisfaction, and caregiver burden would provide a more holistic assessment of success. All-cause mortality was selected as the primary outcome because it was the most consistently reported and objectively defined endpoint across studies, enabling robust quantitative synthesis. Although quality of life is a critically important outcome in populations with limited life expectancy, substantial heterogeneity in measurement tools and reporting, as well as its infrequent reporting, limited its suitability as the primary pooled endpoint. Future deprescribing trials should prioritize standardized patient-reported outcomes alongside survival. Lastly, future reviews should consider equity dimensions, examining how cultural context, socioeconomic status, and healthcare access influence deprescribing decisions and outcomes.

## Conclusion

In synthesis, this comprehensive review affirms that deprescribing preventive medications among frail or end-of-life adults is safe and consistent with best-practice principles of palliative and geriatric medicine. The absence of increased mortality, hospitalization, or major adverse clinical events provides reassurance regarding the safety of deprescribing in this population. Deprescribing should be conceptualized not as withdrawal of care but as refinement of care as an active, evidence-informed process that honors patient autonomy and clinical prudence. By integrating deprescribing into routine clinical pathways, healthcare systems can reduce therapeutic futility, enhance well-being, and uphold the ethical imperative of non-maleficence. As populations age and polypharmacy becomes the norm, such strategies will be essential to align medicine with the meaningful goals of late-life care.

## Supplementary Information

Supplementary Material 1.

## Data Availability

This study is based on publicly available, published data. No individual participant data was generated. The extracted dataset and statistical code used for the meta-analyses are available from the corresponding author upon reasonable request.
