# Supplementary material for: Deprescribing preventive medications in older adults with advanced frailty, dementia, or limited life expectancy: a systematic review and meta-analysis
Source: BMC Geriatr. 2026 Mar 17;26:579. doi: 10.1186/s12877-026-07354-5 (PMC13107709; doi:10.1186/s12877-026-07354-5)
Supplement: Supplementary file 1 — Supplementary Material 1. [file 12877_2026_7354_MOESM1_ESM.docx]

***Supplementary File***

**Table s1.** Full search strategies for all databases.

| **Medline** | | | | | | | | |  |
| --- | --- | --- | --- | --- | --- | --- | --- | --- | --- |
| Interface: **Ovid MEDLINE(R) ALL** content coverage from 1946  Date of Search: October 3, 2025  Number of hits: 2,627  Comment: In Ovid, two or more words are automatically searched as phrases; i.e. no quotation marks are needed | | | | | | Field labels   - exp/ = exploded MeSH term - / = non exploded MeSH term - .ti,ab,kf. = title, abstract and author keywords - adjx = within x words, regardless of order - * = truncation of word for alternate endings - ? = 0-1 letter/number - # = 1 letter/number | | |  |
| Database(s): **Ovid MEDLINE(R) ALL**1946 to October 02, 2025 Search Strategy:   \| **#** \| **Searches** \| **Results** \| \| --- \| --- \| --- \| \| 1 \| Deprescriptions/ \| 1423 \| \| 2 \| Drug Tapering/ \| 579 \| \| 3 \| Drug Utilization Review/ \| 3890 \| \| 4 \| (discontinu* or dis-continu* or deintensif* or de-intensif* or deprescrip* or de-prescrip* or deprescrib* or de-prescrib* or divest* or medica* review* or medica* overview* or tapering or treatment reduction* or withdraw* or withhold*).ti,ab,kf. \| 346625 \| \| 5 \| ((dose? or dosage) adj2 (decreas* or reduce or reducing or reduction*)).ti,ab,kf. \| 60060 \| \| 6 \| (stop* adj3 (drug? or medication* or pescrib* or prescrip* or therap* or treatment*)).ti,ab,kf. \| 20109 \| \| 7 \| or/1-6 \| 419655 \| \| 8 \| Inappropriate Prescribing/ \| 5261 \| \| 9 \| Polypharmacy/ \| 7693 \| \| 10 \| Fibrinolytic Agents/ \| 36055 \| \| 11 \| exp Anticoagulants/ \| 253414 \| \| 12 \| Hydroxymethylglutaryl-CoA Reductase Inhibitors/ \| 37056 \| \| 13 \| Antihypertensive Agents/ \| 75194 \| \| 14 \| exp Hypoglycemic Agents/ or Insulin/ or Metformin/ \| 304572 \| \| 15 \| Platelet Aggregation Inhibitors/ \| 43665 \| \| 16 \| Psychotropic Drugs/ \| 23318 \| \| 17 \| (anticoagula* or anti-coagula* or antidiabetic* or anti-diabetic* or antihyperglycemic* or anti-hyperglycemic* or antihypertensive* or anti-hypertensive* or antiplatelet* or anti-platelet* or antithrombic* or anti-thrombic* or antithrombin* or anti-thrombin* or antithrombotic* or anti-thrombotic* or statin?).ti,ab,kf. \| 353385 \| \| 18 \| (fibrinolytic agent* or fibrinolytic drug* or thrombolytic agent* or thrombolytic drug*).ti,ab,kf. \| 5772 \| \| 19 \| (direct thrombin inhibitor* or factor xa inhibitor* or indirect thrombin inhibitor*).ti,ab,kf. \| 5392 \| \| 20 \| (HMG-CoA reductase inhibitor* or hydroxymethylglutaryl-CoA inhibitor* or hydroxymethylglutaryl CoA reductase inhibitor* or hydroxymethylglutaryl-coenzyme A inhibitor*).ti,ab,kf. \| 5188 \| \| 21 \| (hypoglycemic* or insulin or metformin).ti,ab,kf. \| 477293 \| \| 22 \| (platelet antiaggregant* or platelet aggregation inhibitor* or platelet antagonist* or platelet inhibitor* or protease activated receptor 1 antagonist* or par 1 antagonist*).ti,ab,kf. \| 3576 \| \| 23 \| (psychotropic* or psychoactive agent* or psychoactive drug* or psychopharmaceutical*).ti,ab,kf. \| 26894 \| \| 24 \| (preventive adj3 (drug? or medication*)).ti,ab,kf. \| 3091 \| \| 25 \| (multiple drug therap* or multiple drug treatment* or multidrug therap* or multimedication* or multipharmacotherap* or polypharm* or poly-pharm* or polymedication* or poly-medication* or polypharmacotherap*).ti,ab,kf. \| 18795 \| \| 26 \| (inapprop* adj3 (drug or drugs or medical or medication* or prescrib* or prescrip*)).ti,ab,kf. \| 8944 \| \| 27 \| or/8-26 \| 1212097 \| \| 28 \| Palliative Care/ \| 68081 \| \| 29 \| "Hospice and Palliative Care Nursing"/ \| 2636 \| \| 30 \| Palliative Medicine/ \| 638 \| \| 31 \| Terminal Care/ \| 33967 \| \| 32 \| Hospice Care/ \| 8629 \| \| 33 \| Terminally Ill/ \| 6941 \| \| 34 \| Life Expectancy/ \| 20387 \| \| 35 \| Frailty/ \| 14018 \| \| 36 \| Frail Elderly/ \| 17784 \| \| 37 \| exp Dementia/ \| 231666 \| \| 38 \| Atrial Fibrillation/ \| 79181 \| \| 39 \| Long-Term Care/ \| 29817 \| \| 40 \| Nursing Homes/ \| 41597 \| \| 41 \| ((hospice or palliative or terminal) adj3 (care or healthcare or medicine* or patient* or therap* or treat*)).ti,ab,kf. \| 83289 \| \| 42 \| (end-of-life or "last phase of life").ti,ab,kf. \| 37334 \| \| 43 \| ((before or near) adj death).ti,ab,kf. \| 7148 \| \| 44 \| (life adj2 (expect* or limit*)).ti,ab,kf. \| 55057 \| \| 45 \| ((limit* or poor) adj2 prognos*).ti,ab,kf. \| 164118 \| \| 46 \| (atrial fibrillation* or auricular fibrillation* or debility or debilities or dementia* or frailty or frail elder* or frailness or frailties).ti,ab,kf. \| 316650 \| \| 47 \| ((advanced or terminal*) adj3 (disease* or disorder* or ill or illness)).ti,ab,kf. \| 69936 \| \| 48 \| (long-term adj2 care).ti,ab,kf. \| 34838 \| \| 49 \| ((elder* or geriatr* or nursing or old age) adj3 (facility or facilities or home*)).ti,ab,kf. \| 53370 \| \| 50 \| or/28-49 \| 969918 \| \| 51 \| exp Aged/ or exp Aging/ \| 3967099 \| \| 52 \| (aged or aging or ageing or elder* or geriatr* or sexagenarian* or septuagenarian* or octogenarian* or nonagenarian* or centenarian* or old* age or old* adult* or old* individual* or old* men or old* man or old* patient* or old* people or old* person* or old* population* or old* woman or old* women or oldest old).ti,ab,kf. \| 2009797 \| \| 53 \| or/51-52 \| 5123100 \| \| 54 \| 7 and 27 and 50 and 53 \| 2627 \| | | | | | | | | |  |
| **Embase** | | | | | | | | |  |
| Interface: **embase.com** content coverage from 1947  Date of Search: October 3, 2025  Number of hits: 4,421  Comment: Emtree is the controlled vocabulary in Embase | | | Field labels   - /exp = exploded Emtree term - /de = non exploded Emtree term - ti,ab,kw = title, abstract and author keywords - NEAR/x = within x words, regardless of order - * = truncation of word for alternate endings - $ = 0-1 letter/number - ? = 1 letter/number | | | | | |  |
| \| **Number** \| **Query** \| **Results** \| \| --- \| --- \| --- \| \| #1 \| 'deprescription'/de \| 3015 \| \| #2 \| 'drug dose reduction'/de \| 114145 \| \| #3 \| 'drug utilization review'/de \| 3584 \| \| #4 \| discontinu*:ti,ab,kw OR 'dis continu*':ti,ab,kw OR deintensif*:ti,ab,kw OR 'de intensif*':ti,ab,kw OR deprescrip*:ti,ab,kw OR 'de prescrip*':ti,ab,kw OR deprescrib*:ti,ab,kw OR 'de prescrib*':ti,ab,kw OR divest*:ti,ab,kw OR 'medica* review*':ti,ab,kw OR 'medica* overview*':ti,ab,kw OR tapering:ti,ab,kw OR 'treatment reduction*':ti,ab,kw OR withdraw*:ti,ab,kw OR withhold*:ti,ab,kw \| 575645 \| \| #5 \| (((dose$ OR dosage) NEAR/2 (decreas* OR reduce OR reducing OR reduction*)):ti,ab,kw) OR ((stop* NEAR/3 (drug$ OR medication* OR pescrib* OR prescrip* OR therap* OR treatment*)):ti,ab,kw) \| 145425 \| \| #6 \| #1 OR #2 OR #3 OR #4 OR #5 \| 779945 \| \| #7 \| 'prescribing error'/de \| 7087 \| \| #8 \| 'unnecessary prescribing'/exp \| 1398 \| \| #9 \| 'polypharmacy'/exp \| 30427 \| \| #10 \| 'fibrinolytic agent'/de \| 31473 \| \| #11 \| 'anticoagulant agent'/de OR 'anticoagulant therapy'/de \| 218379 \| \| #12 \| 'hydroxymethylglutaryl coenzyme a reductase inhibitor'/de \| 126983 \| \| #13 \| 'antihypertensive agent'/de OR 'antihypertensive therapy'/de \| 154047 \| \| #14 \| 'antidiabetic agent'/de OR 'insulin'/de OR 'metformin'/de \| 560756 \| \| #15 \| 'antithrombocytic agent'/de \| 62632 \| \| #16 \| 'psychotropic agent'/de \| 40663 \| \| #17 \| anticoagula*:ti,ab,kw OR 'anti coagula*':ti,ab,kw OR antidiabetic*:ti,ab,kw OR 'anti diabetic*':ti,ab,kw OR antihyperglycemic*:ti,ab,kw OR 'anti hyperglycemic*':ti,ab,kw OR antihypertensive*:ti,ab,kw OR 'anti hypertensive*':ti,ab,kw OR antiplatelet*:ti,ab,kw OR 'anti platelet*':ti,ab,kw OR antithrombic*:ti,ab,kw OR 'anti thrombic*':ti,ab,kw OR antithrombin*:ti,ab,kw OR 'anti thrombin*':ti,ab,kw OR antithrombotic*:ti,ab,kw OR 'anti thrombotic*':ti,ab,kw OR statin$:ti,ab,kw \| 576912 \| \| #18 \| 'fibrinolytic agent*':ti,ab,kw OR 'fibrinolytic drug*':ti,ab,kw OR 'thrombolytic agent*':ti,ab,kw OR 'thrombolytic drug*':ti,ab,kw \| 8294 \| \| #19 \| 'direct thrombin inhibitor*':ti,ab,kw OR 'factor xa inhibitor*':ti,ab,kw OR 'indirect thrombin inhibitor*':ti,ab,kw \| 8964 \| \| #20 \| 'hmg-coa reductase inhibitor*':ti,ab,kw OR 'hydroxymethylglutaryl-coa inhibitor*':ti,ab,kw OR 'hydroxymethylglutaryl coa reductase inhibitor*':ti,ab,kw OR 'hydroxymethylglutaryl-coenzyme a inhibitor*':ti,ab,kw \| 7943 \| \| #21 \| hypoglycemic*:ti,ab,kw OR insulin:ti,ab,kw OR metformin:ti,ab,kw \| 688247 \| \| #22 \| ('platelet antiaggregant*':ti,ab,kw OR 'platelet aggregation inhibitor*':ti,ab,kw OR 'platelet antagonist*':ti,ab,kw OR 'platelet inhibitor*':ti,ab,kw OR 'protease activated recept':ti,ab,kw OR #1) AND antagonist*:ti,ab,kw OR 'par 1 antagonist*':ti,ab,kw \| 1200 \| \| #23 \| psychotropic*:ti,ab,kw OR 'psychoactive agent*':ti,ab,kw OR 'psychoactive drug*':ti,ab,kw OR psychopharmaceutical*:ti,ab,kw \| 40941 \| \| #24 \| (preventive NEAR/3 (drug$ OR medication*)):ti,ab,kw \| 4984 \| \| #25 \| 'multiple drug therap*':ti,ab,kw OR 'multiple drug treatment*':ti,ab,kw OR 'multidrug therap*':ti,ab,kw OR multimedication*:ti,ab,kw OR multipharmacotherap*:ti,ab,kw OR polypharm*:ti,ab,kw OR 'poly pharm*':ti,ab,kw OR polymedication*:ti,ab,kw OR 'poly medication*':ti,ab,kw OR polypharmacotherap*:ti,ab,kw \| 30704 \| \| #26 \| (inapprop* NEAR/3 (drug OR drugs OR medical OR medication* OR prescrib* OR prescrip*)):ti,ab,kw \| 14805 \| \| #27 \| #7 OR #8 OR #9 OR #10 OR #11 OR #12 OR #13 OR #14 OR #15 OR #16 OR #17 OR #18 OR #19 OR #20 OR #21 OR #22 OR #23 OR #24 OR #25 OR #26 \| 1712705 \| \| #28 \| 'palliative therapy'/exp \| 163438 \| \| #29 \| 'palliative nursing'/de \| 1912 \| \| #30 \| 'hospice care'/de \| 15196 \| \| #31 \| 'terminally ill patient'/exp \| 10439 \| \| #32 \| 'life expectancy'/de \| 71998 \| \| #33 \| 'frailty'/de \| 42466 \| \| #34 \| 'frail elderly'/de \| 14099 \| \| #35 \| 'dementia'/exp \| 531047 \| \| #36 \| 'atrial fibrillation'/exp \| 263275 \| \| #37 \| 'long term care'/de \| 163235 \| \| #38 \| 'nursing home'/de OR 'nursing home patient'/de \| 77735 \| \| #39 \| ((hospice OR palliative OR terminal) NEAR/3 (care OR healthcare OR medicine* OR patient* OR therap* OR treat*)):ti,ab,kw \| 137793 \| \| #40 \| 'end of life':ti,ab,kw OR 'last phase of life':ti,ab,kw \| 55668 \| \| #41 \| ((before OR near) NEXT/1 death):ti,ab,kw \| 9764 \| \| #42 \| (life NEAR/2 (expect* OR limit*)):ti,ab,kw \| 83761 \| \| #43 \| ((limit* OR poor) NEAR/2 prognos*):ti,ab,kw \| 251683 \| \| #44 \| 'atrial fibrillation*':ti,ab,kw OR 'auricular fibrillation*':ti,ab,kw OR debility:ti,ab,kw OR debilities:ti,ab,kw OR dementia*:ti,ab,kw OR frailty:ti,ab,kw OR 'frail elder*':ti,ab,kw OR frailness:ti,ab,kw OR frailties:ti,ab,kw \| 508497 \| \| #45 \| ((advanced OR terminal*) NEAR/3 (disease* OR disorder* OR ill OR illness)):ti,ab,kw \| 115487 \| \| #46 \| ('long term' NEAR/2 care):ti,ab,kw \| 45398 \| \| #47 \| ((elder* OR geriatr* OR nursing OR 'old age') NEAR/3 (facility OR facilities OR home*)):ti,ab,kw \| 72187 \| \| #48 \| #28 OR #29 OR #30 OR #31 OR #32 OR #33 OR #34 OR #35 OR #36 OR #37 OR #38 OR #39 OR #40 OR #41 OR #42 OR #43 OR #44 OR #45 OR #46 OR #47 \| 1800906 \| \| #49 \| 'aged'/de OR 'aged hospital patient'/de OR 'institutionalized elderly'/de OR 'very elderly'/de OR 'aging'/de \| 4726499 \| \| #50 \| aged:ti,ab,kw OR aging:ti,ab,kw OR ageing:ti,ab,kw OR elder*:ti,ab,kw OR geriatr*:ti,ab,kw OR sexagenarian*:ti,ab,kw OR septuagenarian*:ti,ab,kw OR octogenarian*:ti,ab,kw OR nonagenarian*:ti,ab,kw OR centenarian*:ti,ab,kw OR 'old* age':ti,ab,kw OR 'old* adult*':ti,ab,kw OR 'old* individual*':ti,ab,kw OR 'old* men':ti,ab,kw OR 'old* man':ti,ab,kw OR 'old* patient*':ti,ab,kw OR 'old* people':ti,ab,kw OR 'old* person*':ti,ab,kw OR 'old* population*':ti,ab,kw OR 'old* woman':ti,ab,kw OR 'old* women':ti,ab,kw OR 'oldest old':ti,ab,kw \| 2776323 \| \| #51 \| #49 OR #50 \| 6242422 \| \| #52 \| #6 AND #27 AND #48 AND #51 \| 6790 \| \| #53 \| #52 AND ('Clinical Trial'/it OR 'Conference Abstract'/it) \| 2369 \| \| #54 \| #52 NOT #53 \| 4421 \| | | | | | | | | |  |
| **Cochrane Library** | | | | | | | | |  |
| Interface: **Wiley** content coverage: -Cochrane Database of Systematic Reviews - April 1996 Central Trials - Current content July 1998  Date of Search: October 3, 2025  Number of hits: 779 | | | | | Field labels   - mh = exploded MeSH term - mh ^= non exploded MeSH term - ti,ab,kw = title, abstract and author keywords - NEAR/x = within x words, regardless of order - NEXT = used for truncated phrases - NEXT/x = fixed word order - * = truncation of word for alternate endings - ? = 0-1 letter/number | | | |  |
| \| ID \| Search \| Hits \| \| --- \| --- \| --- \| \| #1 \| [mh ^Deprescriptions] \| 134 \| \| #2 \| [mh ^"Drug Tapering"] \| 73 \| \| #3 \| [mh ^"Drug Utilization Review"] \| 141 \| \| #4 \| (discontinu*:ti,ab,kw OR dis-continu*:ti,ab,kw OR deintensif*:ti,ab,kw OR de-intensif*:ti,ab,kw OR deprescrip*:ti,ab,kw OR de-prescrip*:ti,ab,kw OR deprescrib*:ti,ab,kw OR de-prescrib*:ti,ab,kw OR divest*:ti,ab,kw OR (medica* NEXT review*):ti,ab,kw OR (medica* NEXT overview*):ti,ab,kw OR tapering:ti,ab,kw OR ("treatment" NEXT reduction*):ti,ab,kw OR withdraw*:ti,ab,kw OR withhold*:ti,ab,kw) \| 99623 \| \| #5 \| ((dose?:ti,ab,kw OR dosage:ti,ab,kw) NEAR/2 (decreas*:ti,ab,kw OR reduce:ti,ab,kw OR reducing:ti,ab,kw OR reduction*:ti,ab,kw)) \| 15486 \| \| #6 \| (stop*:ti,ab,kw NEAR/3 (drug?:ti,ab,kw OR medication*:ti,ab,kw OR pescrib*:ti,ab,kw OR prescrip*:ti,ab,kw OR therap*:ti,ab,kw OR treatment*:ti,ab,kw)) \| 6787 \| \| #7 \| #1 OR #2 OR #3 OR #4 OR #5 OR #6 \| 114993 \| \| #8 \| [mh ^"Inappropriate Prescribing"] \| 303 \| \| #9 \| [mh ^Polypharmacy] \| 417 \| \| #10 \| [mh ^"Fibrinolytic Agents"] \| 3432 \| \| #11 \| [mh Anticoagulants] \| 7189 \| \| #12 \| [mh ^"Hydroxymethylglutaryl-CoA Reductase Inhibitors"] \| 5046 \| \| #13 \| [mh ^"Antihypertensive Agents"] \| 10748 \| \| #14 \| [mh "Hypoglycemic Agents"] OR [mh ^Insulin] OR [mh ^Metformin] \| 27442 \| \| #15 \| [mh ^"Platelet Aggregation Inhibitors"] \| 5899 \| \| #16 \| [mh ^"Psychotropic Drugs"] \| 773 \| \| #17 \| (anticoagula*:ti,ab,kw OR anti-coagula*:ti,ab,kw OR antidiabetic*:ti,ab,kw OR anti-diabetic*:ti,ab,kw OR antihyperglycemic*:ti,ab,kw OR anti-hyperglycemic*:ti,ab,kw OR antihypertensive*:ti,ab,kw OR anti-hypertensive*:ti,ab,kw OR antiplatelet*:ti,ab,kw OR anti-platelet*:ti,ab,kw OR antithrombic*:ti,ab,kw OR anti-thrombic*:ti,ab,kw OR antithrombin*:ti,ab,kw OR anti-thrombin*:ti,ab,kw OR antithrombotic*:ti,ab,kw OR anti-thrombotic*:ti,ab,kw OR statin?:ti,ab,kw) \| 68075 \| \| #18 \| (("fibrinolytic" NEXT agent*):ti,ab,kw OR ("fibrinolytic" NEXT drug*):ti,ab,kw OR ("thrombolytic" NEXT agent*):ti,ab,kw OR ("thrombolytic" NEXT drug*):ti,ab,kw) \| 4037 \| \| #19 \| (("direct thrombin" NEXT inhibitor*):ti,ab,kw OR ("factor xa" NEXT inhibitor*):ti,ab,kw OR ("indirect thrombin" NEXT inhibitor*):ti,ab,kw) \| 1726 \| \| #20 \| (("HMG-CoA reductase" NEXT inhibitor*):ti,ab,kw OR ("hydroxymethylglutaryl-CoA" NEXT inhibitor*):ti,ab,kw OR ("hydroxymethylglutaryl CoA reductase" NEXT inhibitor*):ti,ab,kw OR ("hydroxymethylglutaryl-coenzyme A" NEXT inhibitor*):ti,ab,kw) \| 23 \| \| #21 \| (hypoglycemic*:ti,ab,kw OR insulin:ti,ab,kw OR metformin:ti,ab,kw) \| 88580 \| \| #22 \| (("platelet" NEXT antiaggregant*):ti,ab,kw OR ("platelet aggregation" NEXT inhibitor*):ti,ab,kw OR ("platelet" NEXT antagonist*):ti,ab,kw OR ("platelet" NEXT inhibitor*):ti,ab,kw OR ("protease activated receptor 1" NEXT antagonist*):ti,ab,kw OR ("par 1" NEXT antagonist*):ti,ab,kw) \| 6248 \| \| #23 \| (psychotropic*:ti,ab,kw OR ("psychoactive" NEXT agent*):ti,ab,kw OR ("psychoactive" NEXT drug*):ti,ab,kw OR psychopharmaceutical*:ti,ab,kw) \| 3787 \| \| #24 \| (preventive:ti,ab,kw NEAR/3 (drug?:ti,ab,kw OR medication*:ti,ab,kw)) \| 819 \| \| #25 \| (("multiple drug" NEXT therap*):ti,ab,kw OR ("multiple drug" NEXT treatment*):ti,ab,kw OR ("multidrug" NEXT therap*):ti,ab,kw OR multimedication*:ti,ab,kw OR multipharmacotherap*:ti,ab,kw OR polypharm*:ti,ab,kw OR poly-pharm*:ti,ab,kw OR polymedication*:ti,ab,kw OR poly-medication*:ti,ab,kw OR polypharmacotherap*:ti,ab,kw) \| 1914 \| \| #26 \| (inapprop*:ti,ab,kw NEAR/3 (drug:ti,ab,kw OR drugs:ti,ab,kw OR medical:ti,ab,kw OR medication*:ti,ab,kw OR prescrib*:ti,ab,kw OR prescrip*:ti,ab,kw)) \| 1103 \| \| #27 \| #8 OR #9 OR #10 OR #11 OR #12 OR #13 OR #14 OR #15 OR #16 OR #17 OR #18 OR #19 OR #20 OR #21 OR #22 OR #23 OR #24 OR #25 OR #26 \| 162765 \| \| #28 \| [mh ^"Palliative Care"] \| 2672 \| \| #29 \| [mh ^"Hospice and Palliative Care Nursing"] \| 91 \| \| #30 \| [mh ^"Palliative Medicine"] \| 4 \| \| #31 \| [mh ^"Terminal Care"] \| 564 \| \| #32 \| [mh ^"Hospice Care"] \| 182 \| \| #33 \| [mh ^"Terminally Ill"] \| 122 \| \| #34 \| [mh ^"Life Expectancy"] \| 241 \| \| #35 \| [mh ^Frailty] \| 976 \| \| #36 \| [mh ^"Frail Elderly"] \| 1222 \| \| #37 \| [mh Dementia] \| 10137 \| \| #38 \| [mh ^"Atrial Fibrillation"] \| 7554 \| \| #39 \| [mh ^"Long-Term Care"] \| 1538 \| \| #40 \| [mh ^"Nursing Homes"] \| 1990 \| \| #41 \| ((hospice:ti,ab,kw OR palliative:ti,ab,kw OR terminal:ti,ab,kw) NEAR/3 (care:ti,ab,kw OR healthcare:ti,ab,kw OR medicine*:ti,ab,kw OR patient*:ti,ab,kw OR therap*:ti,ab,kw OR treat*:ti,ab,kw)) \| 9886 \| \| #42 \| (end-of-life:ti,ab,kw OR "last phase of life":ti,ab,kw) \| 1861 \| \| #43 \| (end-of-life:ti,ab,kw OR "last phase of life":ti,ab,kw) \| 1861 \| \| #44 \| (life:ti,ab,kw NEAR/2 (expect*:ti,ab,kw OR limit*:ti,ab,kw)) \| 8473 \| \| #45 \| ((limit*:ti,ab,kw OR poor:ti,ab,kw) NEAR/2 prognos*:ti,ab,kw) \| 6519 \| \| #46 \| (("atrial" NEXT fibrillation*):ti,ab,kw OR ("auricular" NEXT fibrillation*):ti,ab,kw OR debility:ti,ab,kw OR debilities:ti,ab,kw OR dementia*:ti,ab,kw OR frailty:ti,ab,kw OR ("frail" NEXT elder*):ti,ab,kw OR frailness:ti,ab,kw OR frailties:ti,ab,kw) \| 42298 \| \| #47 \| ((advanced:ti,ab,kw OR terminal*:ti,ab,kw) NEAR/3 (disease*:ti,ab,kw OR disorder*:ti,ab,kw OR ill:ti,ab,kw OR illness:ti,ab,kw)) \| 7782 \| \| #48 \| (long-term:ti,ab,kw NEAR/2 care:ti,ab,kw) \| 8761 \| \| #49 \| ((elder*:ti,ab,kw OR geriatr*:ti,ab,kw OR nursing:ti,ab,kw OR "old age":ti,ab,kw) NEAR/3 (facility:ti,ab,kw OR facilities:ti,ab,kw OR home*:ti,ab,kw)) \| 7376 \| \| #50 \| #28 OR #29 OR #30 OR #31 OR #32 OR #33 OR #34 OR #35 OR #36 OR #37 OR #38 OR #39 OR #40 OR #41 OR #42 OR #43 OR #44 OR #45 OR #46 OR #47 OR #48 OR #49 \| 87647 \| \| #51 \| [mh Aged] OR [mh Aging] \| 286277 \| \| #52 \| (aged:ti,ab,kw OR aging:ti,ab,kw OR ageing:ti,ab,kw OR elder*:ti,ab,kw OR geriatr*:ti,ab,kw OR sexagenarian*:ti,ab,kw OR septuagenarian*:ti,ab,kw OR octogenarian*:ti,ab,kw OR nonagenarian*:ti,ab,kw OR centenarian*:ti,ab,kw OR (old* NEXT "age"):ti,ab,kw OR (old* NEXT adult*):ti,ab,kw OR (old* NEXT individual*):ti,ab,kw OR (old* NEXT "men"):ti,ab,kw OR (old* NEXT "man"):ti,ab,kw OR (old* NEXT patient*):ti,ab,kw OR (old* NEXT "people"):ti,ab,kw OR (old* NEXT person*):ti,ab,kw OR (old* NEXT population*):ti,ab,kw OR (old* NEXT "woman"):ti,ab,kw OR (old* NEXT "women"):ti,ab,kw OR "oldest old":ti,ab,kw) \| 748191 \| \| #53 \| #51 OR #52 \| 748246 \| \| #54 \| #7 AND #27 AND #50 AND #53 \| 779 \| | | | | | | | | |  |
| **Web of Science Core Collection** | | | | | | | | |  |
| Interface: **Clarivate Analytics**  Editions and content coverage years= A&HCI - 1975 , ESCI -2019 , SCI-EXPANDED - 1945 , SSCI - 1945  Date of Search: October 3, 2025  Number of hits: 1,802 | | Field labels   - TS/Topic = title, abstract, author keywords and Keywords Plus - TI= title - AB = abstract - AK = author keywords - NEAR/x = within x words, regardless of order - * = truncation of word for alternate endings - $ = 0-1 letter/number - ? = 1 letter/number   Note: the *Exact search*-function was used for all the searches | | | | | | |  |
| \| **#** \| **Search Query** \| **Results** \| \| --- \| --- \| --- \| \| 1 \| TS=(discontinu* OR dis-continu* OR deintensif* OR de-intensif* OR deprescrip* OR de-prescrip* OR deprescrib* OR de-prescrib* OR divest* OR "medica* review*" OR "medica* overview*" OR tapering OR "treatment reduction*" OR withdraw* OR withhold* ) \| 559283 \| \| 2 \| TS=((dose$ OR dosage ) NEAR/2 (decreas* OR reduce OR reducing OR reduction* )) \| 85454 \| \| 3 \| TS=(stop* NEAR/3 (drug$ OR medication* OR pescrib* OR prescrip* OR therap* OR treatment* )) \| 20964 \| \| 4 \| #1 OR #2 OR #3 \| 653690 \| \| 5 \| TS=(anticoagula* OR anti-coagula* OR antidiabetic* OR anti-diabetic* OR antihyperglycemic* OR anti-hyperglycemic* OR antihypertensive* OR anti-hypertensive* OR antiplatelet* OR anti-platelet* OR antithrombic* OR anti-thrombic* OR antithrombin* OR anti-thrombin* OR antithrombotic* OR anti-thrombotic* OR statin$ ) \| 424691 \| \| 6 \| TS=("fibrinolytic agent*" OR "fibrinolytic drug*" OR "thrombolytic agent*" OR "thrombolytic drug*" ) \| 5365 \| \| 7 \| TS=("direct thrombin inhibitor*" OR "factor xa inhibitor*" OR "indirect thrombin inhibitor*" ) \| 8100 \| \| 8 \| TS=("HMG-CoA reductase inhibitor*" OR "hydroxymethylglutaryl-CoA inhibitor*" OR "hydroxymethylglutaryl CoA reductase inhibitor*" OR "hydroxymethylglutaryl-coenzyme A inhibitor*" ) \| 6238 \| \| 9 \| TS=(hypoglycemic* OR insulin OR metformin ) \| 685516 \| \| 10 \| TS=("platelet antiaggregant*" OR "platelet aggregation inhibitor*" OR "platelet antagonist*" OR "platelet inhibitor*" OR "protease activated receptor 1 antagonist*" OR "par 1 antagonist*" ) \| 4631 \| \| 11 \| TS=(psychotropic* OR "psychoactive agent*" OR "psychoactive drug*" OR psychopharmaceutical* ) \| 30054 \| \| 12 \| TS=(preventive NEAR/3 (drug$ OR medication* )) \| 3645 \| \| 13 \| TS=("multiple drug therap*" OR "multiple drug treatment*" OR "multidrug therap*" OR multimedication* OR multipharmacotherap* OR polypharm* OR poly-pharm* OR polymedication* OR poly-medication* OR polypharmacotherap* ) \| 22331 \| \| 14 \| TS=(inapprop* NEAR/3 (drug OR drugs OR medical OR medication* OR prescrib* OR prescrip* )) \| 10991 \| \| 15 \| #5 OR #6 OR #7 OR #8 OR #9 OR #10 OR #11 OR #12 OR #13 OR #14 \| 1143566 \| \| 16 \| TS=((hospice OR palliative OR terminal ) NEAR/3 (care OR healthcare OR medicine* OR patient* OR therap* OR treat* )) \| 102031 \| \| 17 \| TS=(end-of-life OR "last phase of life" ) \| 51740 \| \| 18 \| TS=("before death" OR "near death") \| 8026 \| \| 19 \| TS=(life NEAR/2 (expect* OR limit* )) \| 74648 \| \| 20 \| TS=((limit* OR poor ) NEAR/2 prognos* ) \| 182697 \| \| 21 \| TS=("atrial fibrillation*" OR "auricular fibrillation*" OR debility OR debilities OR dementia* OR frailty OR "frail elder*" OR frailness OR frailties ) \| 447482 \| \| 22 \| TS=((advanced OR terminal* ) NEAR/3 (disease* OR disorder* OR ill OR illness )) \| 80036 \| \| 23 \| TS=(long-term NEAR/2 care ) \| 43116 \| \| 24 \| TS=((elder* OR geriatr* OR nursing OR "old age" ) NEAR/3 (facility OR facilities OR home* )) \| 65439 \| \| 25 \| #16 OR #17 OR #18 OR #19 OR #20 OR #21 OR #22 OR #23 OR #24 \| 960122 \| \| 26 \| TS=(aged OR aging OR ageing OR elder* OR geriatr* OR sexagenarian* OR septuagenarian* OR octogenarian* OR nonagenarian* OR centenarian* OR "old* age" OR "old* adult*" OR "old* individual*" OR "old* men" OR "old* man" OR "old* patient*" OR "old* people" OR "old* person*" OR "old* population*" OR "old* woman" OR "old* women" OR "oldest old" ) \| 2326266 \| \| 27 \| #4 AND #15 AND #25 AND #26 \| 1802 \| | | | | | | | | |  |
| **CINAHL** | | | | | | | | | |
| Interface: **EBSCOhost** - content coverage from 1981  Date of Search: October 3, 2025  Number of hits: 715 | | | | Field labels   - MH+ = exploded CINAHL Heading - MH = non exploded CINAHL Heading - TI = title - AB = abstract - XB = title, abstract - Nx = within x words, regardless of order - * = truncation of word for alternate endings - # = 0-1 letter/number - ? = 1 letter/number   Note: sometimes “quotation marks” are needed for single search terms to avoid automatic term mapping (lemmatization) | | | | | |
| \| S# \| Query \| Results \| \| --- \| --- \| --- \| \| S1 \| MH "Deprescribing" \| 671 \| \| S2 \| MH "Drug Tapering" \| 829 \| \| S3 \| XB (discontinu* OR dis-continu* OR deintensif* OR de-intensif* OR deprescrip* OR de-prescrip* OR deprescrib* OR de-prescrib* OR divest* OR "medica* review*" OR "medica* overview*" OR tapering OR "treatment reduction*" OR withdraw* OR withhold* ) \| 72298 \| \| S4 \| XB ((dose# OR dosage ) N2 (decreas* OR reduce OR reducing OR reduction* )) \| 12532 \| \| S5 \| XB (stop* N3 (drug# OR medication* OR pescrib* OR prescrip* OR therap* OR treatment* )) \| 4663 \| \| S6 \| S5 OR S4 OR S3 OR S2 OR S1 \| 87097 \| \| S7 \| MH "Inappropriate Prescribing" \| 4035 \| \| S8 \| MH "Polypharmacy+" \| 6847 \| \| S9 \| MH "Fibrinolytic Agents" \| 8349 \| \| S10 \| MH "Anticoagulants+" \| 35937 \| \| S11 \| MH "Statins" \| 8911 \| \| S12 \| MH "Antihypertensive Agents" \| 16093 \| \| S13 \| MH "Hypoglycemic Agents" \| 19113 \| \| S14 \| MH "Insulin" \| 36027 \| \| S15 \| MH "Metformin" \| 6880 \| \| S16 \| MH "Platelet Aggregation Inhibitors" \| 10384 \| \| S17 \| MH "Psychotropic Drugs" \| 7685 \| \| S18 \| XB (anticoagula* OR anti-coagula* OR antidiabetic* OR anti-diabetic* OR antihyperglycemic* OR anti-hyperglycemic* OR antihypertensive* OR anti-hypertensive* OR antiplatelet* OR anti-platelet* OR antithrombic* OR anti-thrombic* OR antithrombin* OR anti-thrombin* OR antithrombotic* OR anti-thrombotic* OR statin# ) \| 73225 \| \| S19 \| XB ("fibrinolytic agent*" OR "fibrinolytic drug*" OR "thrombolytic agent*" OR "thrombolytic drug*" ) \| 875 \| \| S20 \| XB ("direct thrombin inhibitor*" OR "factor xa inhibitor*" OR "indirect thrombin inhibitor*" ) \| 1184 \| \| S21 \| XB ("HMG-CoA reductase inhibitor*" OR "hydroxymethylglutaryl-CoA inhibitor*" OR "hydroxymethylglutaryl CoA reductase inhibitor*" OR "hydroxymethylglutaryl-coenzyme A inhibitor*" ) \| 686 \| \| S22 \| XB (hypoglycemic* OR insulin OR metformin ) \| 80116 \| \| S23 \| XB ("platelet antiaggregant*" OR "platelet aggregation inhibitor*" OR "platelet antagonist*" OR "platelet inhibitor*" OR "protease activated receptor 1 antagonist*" OR "par 1 antagonist*" ) \| 307 \| \| S24 \| XB (psychotropic* OR "psychoactive agent*" OR "psychoactive drug*" OR psychopharmaceutical* ) \| 6993 \| \| S25 \| XB (preventive N3 (drug# OR medication* )) \| 1114 \| \| S26 \| XB ("multiple drug therap*" OR "multiple drug treatment*" OR "multidrug therap*" OR multimedication* OR multipharmacotherap* OR polypharm* OR poly-pharm* OR polymedication* OR poly-medication* OR polypharmacotherap* ) \| 6137 \| \| S27 \| XB (inapprop* N3 (drug OR drugs OR medical OR medication* OR prescrib* OR prescrip* )) \| 4177 \| \| S28 \| S7 OR S8 OR S9 OR S10 OR S11 OR S12 OR S13 OR S14 OR S15 OR S16 OR S17 OR S18 OR S19 OR S20 OR S21 OR S22 OR S23 OR S24 OR S25 OR S26 OR S27 \| 231678 \| \| S29 \| MH "Palliative Care" OR MH "Palliative Care Nursing" OR MH "Palliative Medicine" \| 46397 \| \| S30 \| MH "Hospice Nursing" OR MH "Hospice Care" \| 15494 \| \| S31 \| MH "Terminal Care+" \| 78509 \| \| S32 \| MH "Terminally Ill Patients" \| 13012 \| \| S33 \| MH "Life Expectancy+" \| 10106 \| \| S34 \| MH "Frailty Syndrome" \| 6824 \| \| S35 \| MH "Frail Elderly" \| 9303 \| \| S36 \| MH "Dementia+" OR MH "Dementia Patients" \| 92669 \| \| S37 \| MH "Atrial Fibrillation" \| 31083 \| \| S38 \| MH "Long Term Care" OR MH "Long Term Care Nursing" \| 29770 \| \| S39 \| MH "Nursing Homes+" \| 32104 \| \| S40 \| XB ((hospice OR palliative OR terminal ) N3 (care OR healthcare OR medicine* OR patient* OR therap* OR treat* )) \| 48398 \| \| S41 \| XB (end-of-life OR "last phase of life" ) \| 26252 \| \| S42 \| XB ((before OR near ) W1 death ) \| 2559 \| \| S43 \| XB (life N2 (expect* OR limit* )) \| 17807 \| \| S44 \| XB ((limit* OR poor ) N2 prognos* ) \| 23417 \| \| S45 \| XB ("atrial fibrillation*" OR "auricular fibrillation*" OR debility OR debilities OR dementia* OR frailty OR "frail elder*" OR frailness OR frailties ) \| 126810 \| \| S46 \| XB ((advanced OR terminal* ) N3 (disease* OR disorder* OR ill OR illness )) \| 20043 \| \| S47 \| XB (long-term N2 care ) \| 23645 \| \| S48 \| XB ((elder* OR geriatr* OR nursing OR "old age" ) N3 (facility OR facilities OR home* )) \| 36542 \| \| S49 \| S48 OR S47 OR S46 OR S45 OR S44 OR S43 OR S42 OR S41 OR S40 OR S39 OR S38 OR S37 OR S36 OR S35 OR S34 OR S33 OR S32 OR S31 OR S30 OR S29 \| 398768 \| \| S50 \| MH "Aged+" AND MH "Aging" \| 30825 \| \| S51 \| XB (aged OR aging OR ageing OR elder* OR geriatr* OR sexagenarian* OR septuagenarian* OR octogenarian* OR nonagenarian* OR centenarian* OR "old* age" OR "old* adult*" OR "old* individual*" OR "old* men" OR "old* man" OR "old* patient*" OR "old* people" OR "old* person*" OR "old* population*" OR "old* woman" OR "old* women" OR "oldest old" ) \| 559457 \| \| S52 \| S50 OR S51 \| 564840 \| \| S53 \| S52 AND S49 AND S28 AND S6 \| 715 \| | | | | | | | | | |
| **ProQuest Dissertations & Theses Global: The Sciences and Engineering Collection** | | | | | | | | | |
| Interface: ProQuest  Content coverage years: from 1673  Date of Search: October 3, 2025  Number of hits: 53 | | | | | | | Field labels   - noft = anywhere except full text - tiabif = title, abstract, keyword - MAINSUBJECT.EXACT = non exploded subject heading - MAINSUBJECT.EXACT.EXPLODE = exploded subject heading - NEAR/x = within x words, regardless of order - * = truncation of word for alternate endings - ? = 1 letter/number   Note: sometimes “quotation marks” are needed for single search terms to avoid automatic term mapping (lemmatization). | | |
| S5 | [[S1] AND [S2] AND [S3] AND [S4]](https://www.proquest.com/recentsearches.recentsearchtabview.recentsearchesgridview.scrolledrecentsearchlist.checkdbssearchlink:rerunsearch/B55AB7282A73463EPQ/None/$N?_csrf=6b92e71e-b0a2-4f16-b4c0-4135fa2880bd&site=pqdtscieng&t:ac=RecentSearches) | | | | | | | 53 | |
| S4 | [(TI,AB,IF(aged) OR TI,AB,IF(aging) OR TI,AB,IF(ageing) OR TI,AB,IF(elder*) OR TI,AB,IF(geriatr*) OR TI,AB,IF(sexagenarian*) OR TI,AB,IF(septuagenarian*) OR TI,AB,IF(octogenarian*) OR TI,AB,IF(nonagenarian*) OR TI,AB,IF(centenarian*) OR TI,AB,IF("old* age") OR TI,AB,IF("old* adult*") OR TI,AB,IF("old* individual*") OR TI,AB,IF("old* men") OR TI,AB,IF("old* man") OR TI,AB,IF("old* patient*") OR TI,AB,IF("old* people") OR TI,AB,IF("old* person*") OR TI,AB,IF("old* population*") OR TI,AB,IF("old* woman") OR TI,AB,IF("old* women") OR TI,AB,IF("oldest old"))](https://www.proquest.com/recentsearches.recentsearchtabview.recentsearchesgridview.scrolledrecentsearchlist.checkdbssearchlink:rerunsearch/2B5CB9A9F42142AFPQ/None/$N?_csrf=6b92e71e-b0a2-4f16-b4c0-4135fa2880bd&site=pqdtscieng&t:ac=RecentSearches) | | | | | | | 115784 | |
| S3 | [((TI,AB,IF(hospice) OR TI,AB,IF(palliative) OR TI,AB,IF(terminal)) NEAR/3 (TI,AB,IF(care) OR TI,AB,IF(healthcare) OR TI,AB,IF(medicine*) OR TI,AB,IF(patient*) OR TI,AB,IF(therap*) OR TI,AB,IF(treat*))) OR (TI,AB,IF(end-of-life) OR TI,AB,IF("last phase of life")) OR (TI,AB,IF("before death") OR TI,AB,IF("near death")) OR (TI,AB,IF(life) NEAR/2 (TI,AB,IF(expect*) OR TI,AB,IF(limit*))) OR ((TI,AB,IF(limit*) OR TI,AB,IF(poor)) NEAR/2 TI,AB,IF(prognos*)) OR (TI,AB,IF("atrial fibrillation*") OR TI,AB,IF("auricular fibrillation*") OR TI,AB,IF(debility) OR TI,AB,IF(debilities) OR TI,AB,IF(dementia*) OR TI,AB,IF(frailty) OR TI,AB,IF("frail elder*") OR TI,AB,IF(frailness) OR TI,AB,IF(frailties)) OR ((TI,AB,IF(advanced) OR TI,AB,IF(terminal*)) NEAR/3 (TI,AB,IF(disease*) OR TI,AB,IF(disorder*) OR TI,AB,IF(ill) OR TI,AB,IF(illness))) OR (TI,AB,IF(long-term) NEAR/2 TI,AB,IF(care)) OR ((TI,AB,IF(elder*) OR TI,AB,IF(geriatr*) OR TI,AB,IF(nursing) OR TI,AB,IF("old age")) NEAR/3 (TI,AB,IF(facility) OR TI,AB,IF(facilities) OR TI,AB,IF(home*)))](https://www.proquest.com/recentsearches.recentsearchtabview.recentsearchesgridview.scrolledrecentsearchlist.checkdbssearchlink:rerunsearch/49F60FEA83274D8DPQ/None/$N?_csrf=6b92e71e-b0a2-4f16-b4c0-4135fa2880bd&site=pqdtscieng&t:ac=RecentSearches) | | | | | | | 37940 | |
| S2 | [(TI,AB,IF(anticoagula*) OR TI,AB,IF(anti-coagula*) OR TI,AB,IF(antidiabetic*) OR TI,AB,IF(anti-diabetic*) OR TI,AB,IF(antihyperglycemic*) OR TI,AB,IF(anti-hyperglycemic*) OR TI,AB,IF(antihypertensive*) OR TI,AB,IF(anti-hypertensive*) OR TI,AB,IF(antiplatelet*) OR TI,AB,IF(anti-platelet*) OR TI,AB,IF(antithrombic*) OR TI,AB,IF(anti-thrombic*) OR TI,AB,IF(antithrombin*) OR TI,AB,IF(anti-thrombin*) OR TI,AB,IF(antithrombotic*) OR TI,AB,IF(anti-thrombotic*) OR TI,AB,IF(statin?)) OR (TI,AB,IF("fibrinolytic agent*") OR TI,AB,IF("fibrinolytic drug*") OR TI,AB,IF("thrombolytic agent*") OR TI,AB,IF("thrombolytic drug*")) OR (TI,AB,IF("direct thrombin inhibitor*") OR TI,AB,IF("factor xa inhibitor*") OR TI,AB,IF("indirect thrombin inhibitor*")) OR (TI,AB,IF("HMG-CoA reductase inhibitor*") OR TI,AB,IF("hydroxymethylglutaryl-CoA inhibitor*") OR TI,AB,IF("hydroxymethylglutaryl CoA reductase inhibitor*") OR TI,AB,IF("hydroxymethylglutaryl-coenzyme A inhibitor*")) OR (TI,AB,IF(hypoglycemic*) OR TI,AB,IF(insulin) OR TI,AB,IF(metformin)) OR (TI,AB,IF("platelet antiaggregant*") OR TI,AB,IF("platelet aggregation inhibitor*") OR TI,AB,IF("platelet antagonist*") OR TI,AB,IF("platelet inhibitor*") OR TI,AB,IF("protease activated recept") OR 1 TI,AB,IF(antagonist*) OR TI,AB,IF("par 1 antagonist*")) OR (TI,AB,IF(psychotropic*) OR TI,AB,IF("psychoactive agent*") OR TI,AB,IF("psychoactive drug*") OR TI,AB,IF(psychopharmaceutical*)) OR (TI,AB,IF(preventive) NEAR/3 (TI,AB,IF(drug?) OR TI,AB,IF(medication*))) OR (TI,AB,IF("multiple drug therap*") OR TI,AB,IF("multiple drug treatment*") OR TI,AB,IF("multidrug therap*") OR TI,AB,IF(multimedication*) OR TI,AB,IF(multipharmacotherap*) OR TI,AB,IF(polypharm*) OR TI,AB,IF(poly-pharm*) OR TI,AB,IF(polymedication*) OR TI,AB,IF(poly-medication*) OR TI,AB,IF(polypharmacotherap*)) OR (TI,AB,IF(inapprop*) NEAR/3 (TI,AB,IF(drug) OR TI,AB,IF(drugs) OR TI,AB,IF(medical) OR TI,AB,IF(medication*) OR TI,AB,IF(prescrib*) OR TI,AB,IF(prescrip*)))](https://www.proquest.com/recentsearches.recentsearchtabview.recentsearchesgridview.scrolledrecentsearchlist.checkdbssearchlink:rerunsearch/F6E1988D06FF4A0BPQ/None/$N?_csrf=6b92e71e-b0a2-4f16-b4c0-4135fa2880bd&site=pqdtscieng&t:ac=RecentSearches) | | | | | | | 55917 | |
| S1 | [(TI,AB,IF(discontinu*) OR TI,AB,IF(dis-continu*) OR TI,AB,IF(deintensif*) OR TI,AB,IF(de-intensif*) OR TI,AB,IF(deprescrip*) OR TI,AB,IF(de-prescrip*) OR TI,AB,IF(deprescrib*) OR TI,AB,IF(de-prescrib*) OR TI,AB,IF(divest*) OR TI,AB,IF("medica* review*") OR TI,AB,IF("medica* overview*") OR TI,AB,IF(tapering) OR TI,AB,IF("treatment reduction*") OR TI,AB,IF(withdraw*) OR TI,AB,IF(withhold*)) OR ((TI,AB,IF(dose?) OR TI,AB,IF(dosage)) NEAR/2 (TI,AB,IF(decreas*) OR TI,AB,IF(reduce) OR TI,AB,IF(reducing) OR TI,AB,IF(reduction*))) OR (TI,AB,IF(stop*) NEAR/3 (TI,AB,IF(drug?) OR TI,AB,IF(medication*) OR TI,AB,IF(pescrib*) OR TI,AB,IF(prescrip*) OR TI,AB,IF(therap*) OR TI,AB,IF(treatment*)))](https://www.proquest.com/recentsearches.recentsearchtabview.recentsearchesgridview.scrolledrecentsearchlist.checkdbssearchlink:rerunsearch/978384EC5F9A4122PQ/None/$N?_csrf=6b92e71e-b0a2-4f16-b4c0-4135fa2880bd&site=pqdtscieng&t:ac=RecentSearches) | | | | | | | 42157 | |

**Table s2.** Results of the risk of bias assessment of the included randomized controlled trials using the revised Cochrane risk-of-bias 2 tool.

| **Author, year** | **Bias arising from the randomization process** | **Bias due to deviations from intended interventions** | **Bias due to missing outcome data** | **Bias due to measurement of the outcome** | **Bias in selection of the reported result** | **Overall bias** |
| --- | --- | --- | --- | --- | --- | --- |
| Benetos, 2025 | Low | Low | Low | Low | Low | Low |
| Curtin, 2020 | Low | Moderate | Low | Moderate | Low | Moderate |
| Gulla, 2018 | Moderate | Moderate | Low | Moderate | Low | Moderate |
| Husebø, 2019 | Low | Moderate | Low | Low | Low | Moderate |
| Kutner, 2015 | Low | Low | Low | Low | Low | Low |
| Moonen, 2016 | Low | Low | Low | Low | Low | Low |
| Mortsiefer, 2023 | Low | Moderate | Low | Low | Low | Moderate |
| Potter, 2016 | Low | Moderate | Low | Moderate | Low | Moderate |
| Sheppard, 2020 | Low | Low | Low | Low | Low | Low |
| Sheppard, 2024 | Low | Low | Low | Low | Low | Low |

**Table s3.** Results of the risk of bias assessment of the included observational studies using the ROBINS-I tool.

| **Studies** | **Bias due to confounding** | **Bias in selection of participants into the study** | **Bias in classification of interventions** | **Bias due to deviations from intended interventions** | **Bias due to missing data** | **Bias in measurement of outcomes** | **Bias in selection of the reported result** | **Overall bias** |
| --- | --- | --- | --- | --- | --- | --- | --- | --- |
| Bertozzo, 2016 | Moderate | Low | Low | Low | Low | Low | Low | Moderate |
| Chin-Yee, 2022 | Moderate | Low | Low | Low | Low | Low | Low | Moderate |
| Ioffe, 2021 | Serious | Moderate | Low | Low | Low | Low | Moderate | Serious |
| Kempers, 2025 | Moderate | Low | Low | Low | Low | Low | Low | Moderate |
| Niznik, 2022 | Low | Low | Low | Low | Low | Low | Low | Low |


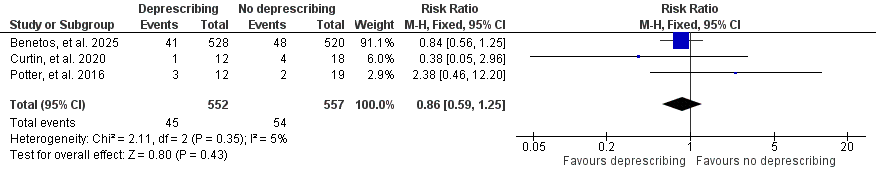


**Figure s1.** Forest plot showing the effect of deprescribing vs. no deprescribing of various preventive medications on fractures in older adults with frailty or limited life expectancy.


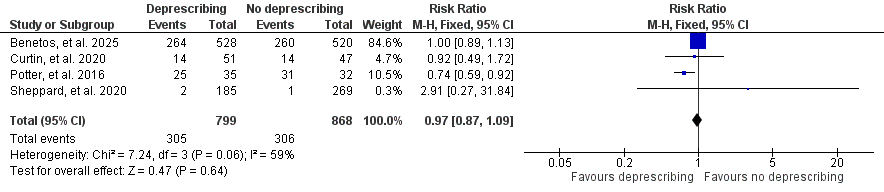


**Figure s2.** Forest plot showing the effect of deprescribing vs. no deprescribing of various preventive medications on falls in older adults with frailty or limited life expectancy.


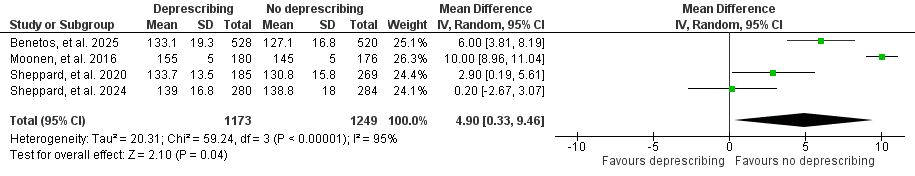


**Figure s3.** Forest plot showing the effect of deprescribing vs. no deprescribing of antihypertensives on systolic blood pressure in older adults with frailty or limited life expectancy.


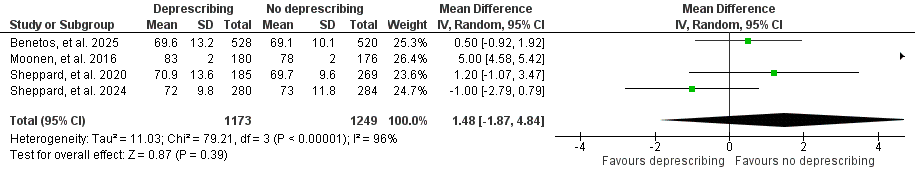


**Figure s4.** Forest plot showing the effect of deprescribing vs. no deprescribing of antihypertensives on systolic blood pressure in older adults with frailty or limited life expectancy.


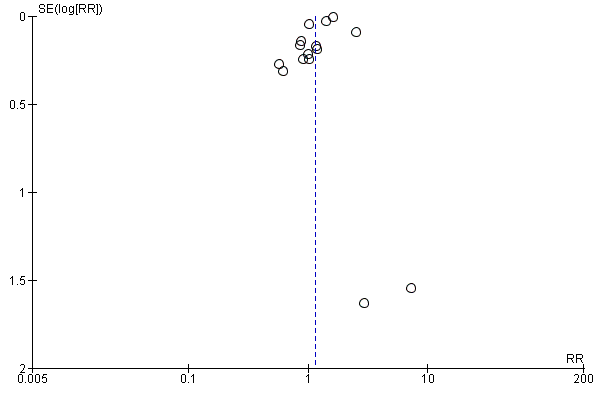


**Figure s5.** Funnel plot showing the effect of deprescribing vs. no deprescribing on all-cause mortality in older adults with frailty or limited life expectancy.


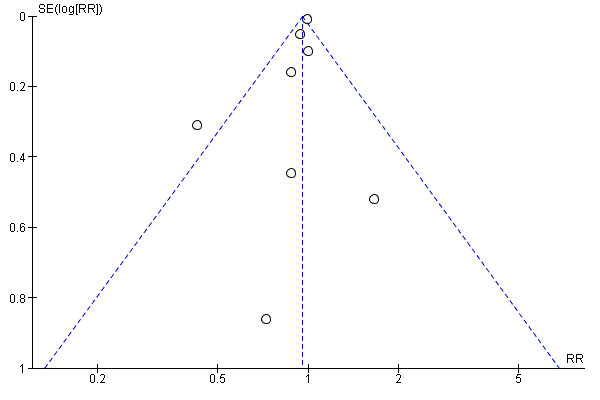


**Figure s6.** Funnel plot showing the effect of deprescribing vs. no deprescribing on hospitalization in older adults with frailty or limited life expectancy.


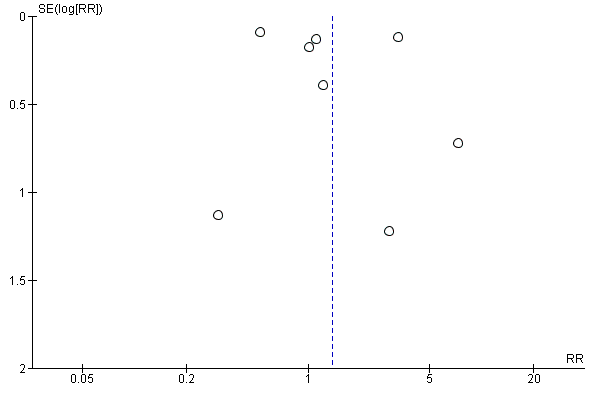


**Figure s7.** Funnel plot showing the effect of deprescribing vs. no deprescribing on major adverse cardiovascular events, including thromboembolic events in older adults with frailty or limited life expectancy.

**Table s4.** Certainty of evidence (GRADE).

| **Certainty assessment** | | | | | | | | **No. of patients** | | | **Effect** | | **Certainty** | **Importance** |  |
| --- | --- | --- | --- | --- | --- | --- | --- | --- | --- | --- | --- | --- | --- | --- | --- |
| **No. of studies** | **Study design** | **Risk of bias** | **Inconsistency** | **Indirectness** | **Imprecision** | **Other considerations** | **Deprescribing preventive medications** | | **Continuation of preventive medicines** | **Relative (95% CI)** | | **Absolute (95% CI)** |  |  |  |
| **All-cause mortality** | | | | | | | | | | | | | | | |
| 15 | randomized trial and non-randomised studies | serious^a^ | not serious | not serious | not serious | none | 4944/8933 (55.3%) | | 10240/24925 (41.1%) | **RR 1.15** (0.98 to 1.35) | | **62 more per 1,000** (from 8 fewer to 144 more) | ⨁◯◯◯ Very low^a^ | CRITICAL |  |
| **Hospitalization** | | | | | | | | | | | | | | | |
| 8 | randomized trial and non-randomised studies | serious^b^ | not serious | not serious | not serious | none | 618/1711 (36.1%) | | 822/2715 (30.3%) | **RR 0.93** (0.82 to 1.07) | | **21 fewer per 1,000** (from 54 fewer to 21 more) | ⨁◯◯◯ Very low^b^ | IMPORTANT |  |
| **Major adverse cardiovascular events** | | | | | | | | | | | | | | | |
| 8 | randomized trial and non-randomised studies | serious^c^ | not serious | not serious | not serious | none | 429/7285 (5.9%) | | 1139/22318 (5.1%) | **RR 1.37** (0.70 to 2.70) | | **19 more per 1,000** (from 15 fewer to 87 more) | ⨁◯◯◯ Very low^c^ | IMPORTANT |  |
| **Quality of life** | | | | | | | | | | | | | | | |
| 6 | randomized trial and non-randomised studies | serious^d^ | not serious | not serious | not serious | none | 930 | | 961 | - | | SMD **0.1 SD lower** (0.31 lower to 0.11 higher) | ⨁◯◯◯ Very low^d^ | IMPORTANT |  |

CI: confidence interval; RR: relative risk; SMD: standardized mean difference.

^a.^ RCTs; 5 out of 10 had a moderate risk of bias; observational studies: 3 out of 5 had a moderate risk of bias, and 1 out of 5 had a serious risk of bias.

^b.^ RCTs; 4 out of 7 had a moderate risk of bias.

^c.^ RCTs; 1 out of 5 had a moderate risk of bias; observational studies: 3 out of 3 had a moderate risk of bias.

^d.^ RCTS: 3 out of 6 had a moderate risk of bias.
